# From [^99m^Tc]pertechnetate to [^99m^Tc]sestamibi: Dissection of a Complex Reaction Sequence Using Radio-LC-MS

**DOI:** 10.3390/molecules31040596

**Published:** 2026-02-09

**Authors:** Joana do Mar Ferreira Machado, Antonio Shegani, Ingebjørg N. Hungnes, Truc T. Pham, Amaia Carrascal-Miniño, Margaret S. Cooper, Victoria Gibson, Levente K. Meszaros, Michelle T. Ma, Philip J. Blower

**Affiliations:** 1 School of Biomedical Engineering and Imaging Sciences, St Thomas’ Hospital, King’s College London, 4th Floor Lambeth Wing, London SE1 7EH, UK; 2 Department of Nuclear Medicine, Guy’s and St Thomas’ NHS Foundation Trust, London SE1 9RT, UK; 3 Nanomab Technology (UK) Limited, Elstree, Hertfordshire WD6 3SY, UK

**Keywords:** technetium, MIBI, sestamibi, radiopharmaceutical, methoxyisobutylisonitrile, LC-MS, mass spectrometry

## Abstract

[^99m^Tc]sestamibi ([^99m^Tc][Tc(MIBI)_6_]^+^; MIBI = 2-methoxybutylisonitrile) is a clinically established myocardial perfusion SPECT tracer. Its one-pot kit-based synthesis from [^99m^Tc]pertechnetate ([^99m^Tc][TcO_4_]^−^) is complex, involving a 6-oxidation state transition (Tc(VII) to Tc(I)) and complete ligand replacement. We aimed to unravel this complex reaction, to inform rational quality control and identify new technetium synthons for molecular imaging. Generator-produced [^99m^Tc]pertechnetate was added to commercial or bespoke clinically used kits, varying the reaction time, temperature, and concentrations of reagents (individually and collectively) and carrier technetium-99. Radioactive products were analysed by thin-layer chromatography (TLC) and high-performance liquid chromatography (HPLC) with optical, radiometric, and mass spectrometric (MS-ESI+) detection. At least 11 radioactive intermediates were detected by radio-HPLC. Technetium(V) and technetium(I) intermediates were identified or imputed by radio-HPLC-MS, including [Tc^V^O(cysteinate)_2_]^+^, [Tc^I^(MIBI)_4_L_2_]^+^, and [Tc^I^(MIBI)_5_L_1_]^+^ (L = labile monodentate ligand, e.g., H_2_O). Tc(III) intermediates [Tc^III^(cysteinate)_2_(MIBI)]^+^ and [Tc^III^(cysteinate)_2_(MIBI)_2_]^+^ were indicated by weak MS-ESI+ ions. We conclude that the reaction proceeds via reduction from [Tc^VII^O_4_]^−^ via unknown intermediates to [Tc^V^O(cysteinate)_2_]^+^, then via Tc(III) intermediates containing both cysteinate and MIBI ligands (e.g., [Tc^III^(cysteinate)_2_(MIBI)_2_]^+^), to form Tc(I) without cysteine and with <6 MIBI ligands, followed by further ligand displacement by MIBI to form [Tc(MIBI)_6_]^+^. Once formed, [Tc(MIBI)_6_]^+^ undergoes no further reaction.

## 1. Introduction

**Figure 1:**
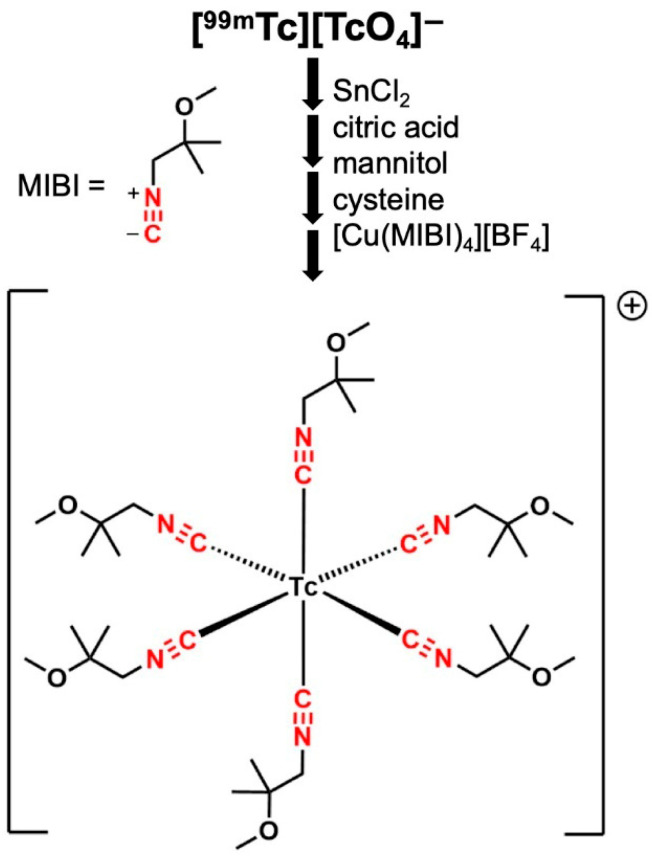
Structure of [Tc(MIBI)_6_]^+^ and reagents involved in its one-pot, multi-step synthesis from [^99m^Tc][TcO_4_]^−^ via unknown intermediates.

[^99m^Tc]sestamibi ([^99m^Tc][Tc(MIBI)_6_]^+^; MIBI = methoxyisobutylisonitrile, Figure 1) is a radiopharmaceutical established in clinical use for more than three decades for myocardial perfusion scintigraphy or single-photon tomography. It is a cationic Tc(I) complex, developed in the 1980s [1,2,3] in a search for cationic technetium complexes to replace the myocardial perfusion imaging tracer [^201^Tl]TlCl. It received FDA approval in 1990. Although its myocardial perfusion imaging application is based on early recognition of its uptake and trapping in myocardium in proportion to blood flow, it was later discovered that the trapping mechanism was uptake in mitochondria [4] in proportion to the mitochondrial membrane potential, for which its key chemical characteristics are its cationic charge (providing a thermodynamic basis for accumulation in the negatively charged mitochondrial matrix) and its lipophilicity, which allows it to penetrate the plasma and mitochondrial membranes. This property was later shown to have the potential to image mitochondrial toxicity [5]. It was also found to be useful in parathyroid [6] and cancer [7] imaging. The discovery that it is a substrate for P-glycoprotein [8,9] generated applications in predicting multidrug resistance in cancer.

The complex is prepared for the clinic in a one-pot synthesis using a commercially produced kit—originally “Cardiolite”, latterly also “Technescan MIBI” and “Stamicis”—to which [^99m^Tc]pertechnetate is added in the form of ^99^Mo/^99m^Tc-generator eluate in saline, followed by heating at 100 °C for 10 min. The kit contains [Cu(MIBI)_4_][BF_4_], sodium citrate dihydrate, L-cysteine hydrochloride monohydrate, mannitol, and stannous chloride dihydrate [10]. The reaction is necessarily complex, involving a six-electron reduction of technetium from Tc(VII) to Tc(I) as well as complete replacement of the oxygen ligands, and presumably proceeds via multiple intermediates, none of which have been characterised in the four decades since its discovery. Even the final product was identified as [^99m^Tc][Tc(MIBI)_6_]^+^ by inference from the more complete characterisation of other technetium(I) hexakis(isonitrile) complexes [11]; its identity was confirmed later by mass spectrometry of a reconstituted kit [12]. A few reports mention a small number of intermediates or very low-level impurities after variation in the standard reaction conditions, detected by radio-high-performance liquid chromatography (HPLC) without analysis or comment on their identity [13]. A technetium–cysteine complex has been suggested as an intermediate, without spectroscopic or other evidence [10]. Potential intermediate species [^99m^Tc][Tc(MIBI)_4_]^+^ (*m*/*z* = 551) and [^99m^Tc][Tc(MIBI)_5_]^+^ (*m*/*z* = 664) were detected by mass spectrometry [14], but no systematic study of the reaction sequence has been reported.

**Figure 2:**
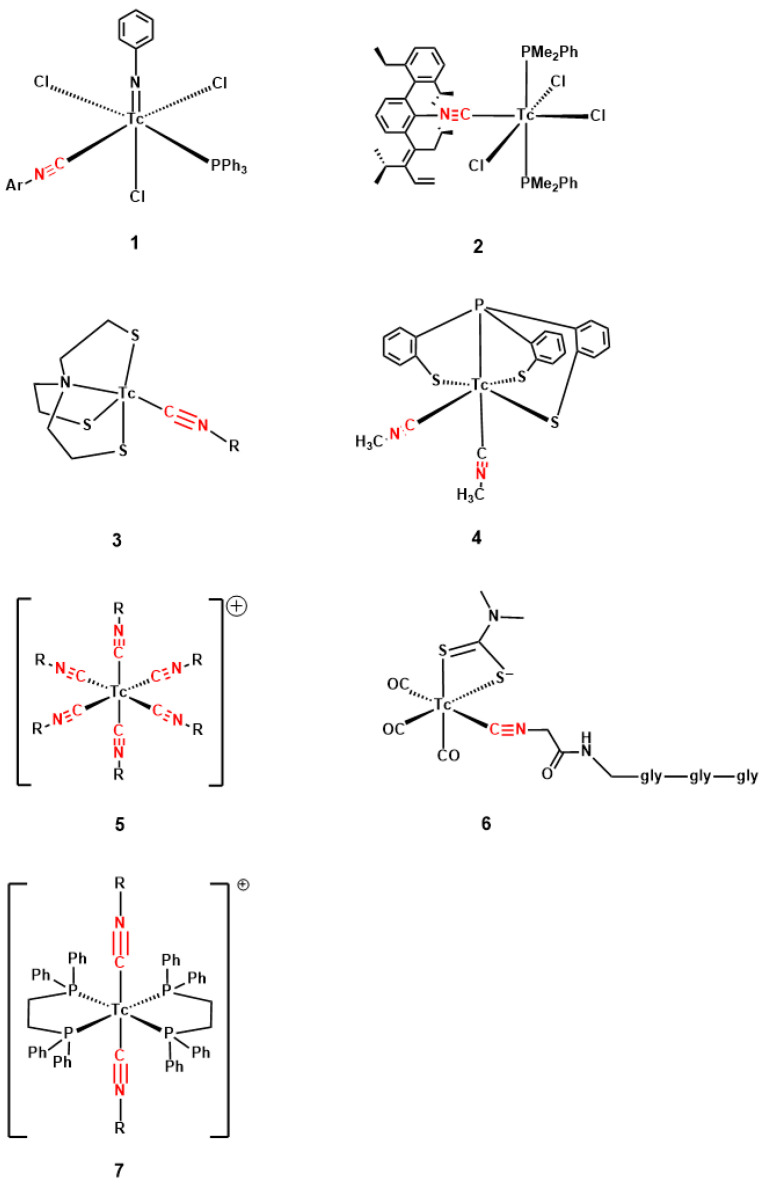
Structures of known technetium isonitrile complexes containing Tc(V) (**1**); Tc(III) (**2**, **3**, **4**); and Tc(I) (**5**, **6**, **7**).

Reports of technetium isonitrile coordination chemistry have been limited so far to a few complexes, in which the isonitrile coordinates Tc in oxidation states V, III, or I (Figure 2). Tc(V) complexes are represented only by the phenylimido complex **1** (Figure 2) [15]. Nitrido complexes containing MIBI have been reported, but not structurally characterised [16]. Tc(III) isonitrile complexes with halide and tertiary phosphine co-ligands (**2**, Figure 2) have been synthesised by the substitution of a phosphine for an isonitrile in [TcCl_3_(PMe_2_Ph)_3_] [17]. Tetradentate amino- and phosphino-trithiolate “umbrella” ligands support a Tc(III) binding site for one or two isonitrile ligands (**3** and **4**, Figure 2) [18,19,20]. Tc(I) has the richest reported range of isonitrile complexes, as might be expected given the strong pi-acceptor character of isonitriles. The pi-accepting ligand set stabilising Tc(I) may be provided exclusively by isonitriles ([Tc(CNR)_6_]^+^ (**5** in Figure 2 [21], exemplified by [Tc(MIBI)_6_]^+^); these complexes are accessible by ligand substitution and the reduction of Tc(V) [3,11,15] or Tc(III) [21], with isonitrile serving as a reducing agent, or by the reduction of pertechnetate with dithionite in the presence of the isonitrile [1,2]. Alternatively, the isonitrile ligands may be accompanied by other pi-accepting ligands—the tricarbonyl ligand set (e.g., **6** in Figure 2, accompanied here by a dithiocarbamate ligand [22]) or tertiary phosphines, as in **7** in Figure 2 [23].

In this paper, we report efforts—the most detailed to date, updating preliminary reports [24,25]—to understand the obscure and complex process by which [^99m^Tc][TcO_4_]^−^ is converted to the [^99m^Tc][Tc(MIBI)_6_]^+^ complex using this kit. We employed HPLC with three detection modalities operating in series—UV optical detection, gamma-ray detection, and electrospray mass spectrometry (MS-ES+)—and interpreted the results in light of the rather sparse background of known and characterised technetium isonitrile complexes.

## 2. Results

### 2.1. Radiochromatography

The standard preparation of the [^99m^Tc][Tc(MIBI)_6_]^+^ complex involves reconstituting a kit vial with ^99m^Tc generator eluate and incubating at 100 °C for 10 min. As an initial demonstration of the complexity of the reaction pathway from [TcO_4_]^−^ (Tc(VII)) to [Tc(MIBI)_6_]^+^ (Tc(I)), and to assist with detecting intermediates, changes to the reaction conditions including reducing temperature, varying incubation time, reducing concentration of kit components, increasing the concentration of technetium by adding carrier [NH_4_][^99^TcO_4_], and removing individual chemical components, were evaluated. Radiochemical analysis of products was performed using thin-layer chromatography (TLC) and reversed-phase HPLC. Unreacted [^99m^Tc][TcO_4_]^−^ and [^99m^Tc][Tc(MIBI)_6_]^+^ prepared under standard conditions (100 °C, 10 min) were analysed similarly for comparison.

TLC methods are detailed in the Supplementary Information, Table S1. Using TLC method 1 (stationary phase: alumina; mobile phase: ethanol), “standard” [^99m^Tc][Tc(MIBI)_6_]^+^ showed a single dominant peak with Rf = 0.82, with a very small Rf = 0 peak assigned to “reduced-hydrolysed technetium”. [^99m^Tc][TcO_4_]^−^ showed a broad peak, with Rf = 0.1–0.5 (Figure S1). TLC of the same samples using method 2 (stationary phase: iTLC-SG; mobile phase: saline) showed a single peak with Rf < 0.1 for [^99m^Tc][Tc(MIBI)_6_]^+^ (including the trace of reduced-hydrolysed technetium identified in method 1) and a single peak with Rf > 0.9 for [^99m^Tc][TcO_4_]^−^ (Figure S2).

**Figure 3:**
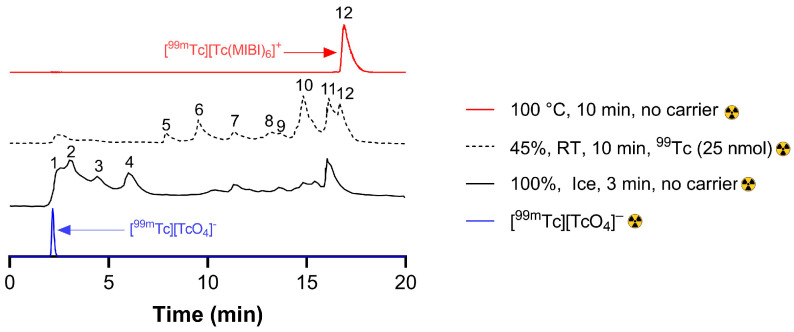
Exemplar radiochromatograms showing main intermediates produced under a range of temperatures, incubation times, kit dilutions (% of kit content used), and added ^99^Tc carrier concentrations, showing peak labelling scheme and progression from hydrophilic early-forming intermediates (peaks 1–4), through moderately lipophilic intermediates (peaks 5–9), to highly lipophilic late-forming intermediates (peaks 10–11) and final product [^99m^Tc][Tc(MIBI)_6_]^+^ (peak 12).

Reverse-phase radio-HPLC (method 1, a water/methanol/formic acid gradient, Table S2) of standard [^99m^Tc][Tc(MIBI)_6_]^+^ showed a dominant single peak eluting at 16.9 min, with trace radiochemical impurities at 2.2–2.5 min (<1%), while [^99m^Tc][TcO_4_]^−^ directly from a generator eluted as a very sharp peak at 2.2 min (Figure S3). Incubating at room temperature or on ice for 0.5–60 min (see Table S3) gave a much more complex series of radiochromatograms, with at least 12 distinguishable peaks (Figure S4). Early in the incubation (0.5–1.5 min), the chromatogram was dominated by hydrophilic species eluting between 2 and 7 min; later in the incubation, a series of smaller peaks eluted between 7 and 13 min, and later, still more lipophilic species appeared, eluting between 13 and 18 min. By 60 min, the radiochromatogram approached that of “standard” [^99m^Tc][Tc(MIBI)_6_]^+^, with a residual minor impurity at ca. 16 min. Further cooling to 0 °C (Table S3) produced a similar number of peaks with similar elution times but different abundances, indicating the same intermediates but a still slower reaction (Figure S5); even at 60 min, the chromatogram did not approach that of “standard” [^99m^Tc][Tc(MIBI)_6_]^+^ (Figure S5). A summary of the HPLC chromatograms obtained by these variations in conditions is shown in Figure 3. Size exclusion chromatography resolved fewer intermediates (see Figure S6) and was not pursued further.

Dilution of the cold kit contents by fractionation (Table S4) also slowed the room temperature reactions to produce radio-HPLC profiles (Figure S7) with elution times similar to those in Figure 3. Dilution-induced slowing was accompanied by increasing radioactivity trapped in the HPLC column, presumably due to increased reduced-hydrolysed technetium (Table S5).

Adding carrier [NH_4_][^99^TcO_4_] (Table S6), while reducing the concentration of kit components, allowed kit components—especially stannous chloride—rather than pertechnetate to become rate-limiting, offering additional ways to enhance the profile of selected intermediates and increasing the technetium concentration to improve prospects of identifying species by mass spectrometry (vide infra), where sensitivity is challenging. Importantly, in this context, while the relative abundance of radioactive peaks could be controlled, their elution times were not significantly altered. For example, Figures S8 and S9 show how the progression of the reaction was slowed by a progressive increase in added ^99^Tc, and more so when the non-radioactive kit components were diluted, allowing later-eluting peaks to dominate at low technetium concentrations and early-eluting peaks to dominate at higher technetium concentrations. Peaks 1–3 dominated (Figure 3) after 0.5 min incubation at 0 °C with moderate dilution and added carrier (condition 25, Table S6); peak 4 dominated after 5 min at room temperature with moderate carrier (condition 3); peaks 5–11 could be enhanced by using condition 15 (incubation time 10 min); and peak 12 ([^99m^Tc][Tc(MIBI)_6_]^+^) dominated under heating at 100 °C for 10 min.

To assess the contribution of MIBI itself to the various intermediates, bespoke kit vials were assembled mimicking the standard kit but with [Cu(MIBI)_4_][BF_4_] reduced or absent (kits 1–4; Table S7). After reconstitution and heating to 100 °C for 10 min, HPLC chromatograms showed delayed and incomplete conversion of the hydrophilic species into the more lipophilic intermediates; kit 1, containing no [Cu(MIBI)_4_][BF_4_], showed no radioactivity eluting after 7 min (Figure S10), suggesting that MIBI is essential to form species eluting >7 min but plays no part in formation of the early-forming hydrophilic intermediates (1–4). This is consistent with the more lipophilic intermediates appearing later in the reaction.

The bespoke kits, 5–8, were prepared without [Cu(MIBI)_4_][BF_4_] and one or more of the other kit components; stannous chloride was present in all of them (Table S8). They produced chromatograms with no peaks eluting after 7 min (Figure S11). Kit 5, containing only citrate and stannous chloride, transitioned through intermediates eluting between 2.1 and 3.5 min, producing a single sharp peak at 2.2 min, which was not readily distinguishable from pertechnetate. Kit 8 (containing only mannitol and stannous chloride) produced a single peak eluting at 2.3 min, again not conclusively distinguishable from [^99m^Tc][TcO_4_]^−^. Kits 6 (containing only cysteine and mannitol) and 7 (containing only cysteine) both produced a single sharp peak eluting at 5.2 min (corresponding to peak 4 in Figure 2), via an intermediate eluting at 1.9 min (again, not conclusively distinguishable from pertechnetate). These results suggest that the species eluting around 5–6 min (peak 4) is a cysteine complex, while the species eluting immediately before and after [^99m^Tc][TcO_4_]^−^ (peaks 1–3) are citrate complexes. Resolution of these early-eluting radioactive species is not adequate to draw firm conclusions about the role of mannitol.

Suspecting that some of the more persistent intermediates in the HPLC radiochromatogram might have sufficient kinetic stability to be isolated for further analysis, attempts were made to separate them by HPLC and to re-analyse the purified fractions. These results are presented in Supplementary Figures S19–S22.

By means of the experiments described above, a set of reaction and HPLC conditions, exemplified in Figure 3, was established for use in subsequent LC-MS studies to identify chemical compositions of the intermediates. In some cases, radio-TLC of the products was performed and compared with radio-HPLC, showing that TLC was not capable of distinguishing intermediates, and, importantly, that some samples producing a TLC chromatogram deemed suitable for release (radiochemical purity > 90%) in fact contained less than 30% [^99m^Tc][Tc(MIBI)_6_]^+^, as measured by HPLC method 1. An example is shown in Figure S23.

### 2.2. LCMS

Electrospray mass spectrometry is one of the few radioactivity-independent analytical techniques with sufficient sensitivity to detect the very low concentrations of radioactive analytes. By incorporating MS-ESI+ in-line as part of a triple-modality HPLC detector system comprising, serially, optical (UV-visible), radioactivity (gamma), and MS-ESI+, it was possible to use the radiochromatogram to focus MS-ESI+ analysis on the radioactive (and hence technetium-containing) fractions of the HPLC eluate, and to analyse each radioactive intermediate (peaks 1–12) individually. Since some non-radioactive kit components would inevitably co-elute with some radioactive species, kits to which no technetium had been added were analysed so that non-technetium-containing species (mannitol, Figure S12; citric acid, Figure S13; cysteine, Figure S14; [Cu(MIBI)_4_][BF_4_], Figures S15 and S16) could be eliminated. No additional peaks were detected when Technescan MIBI kits (Mallinckrodt Pharmaceuticals, Petten, The Netherlands) were subjected to the same analysis after reconstitution with saline or water.

**Figure 4:**
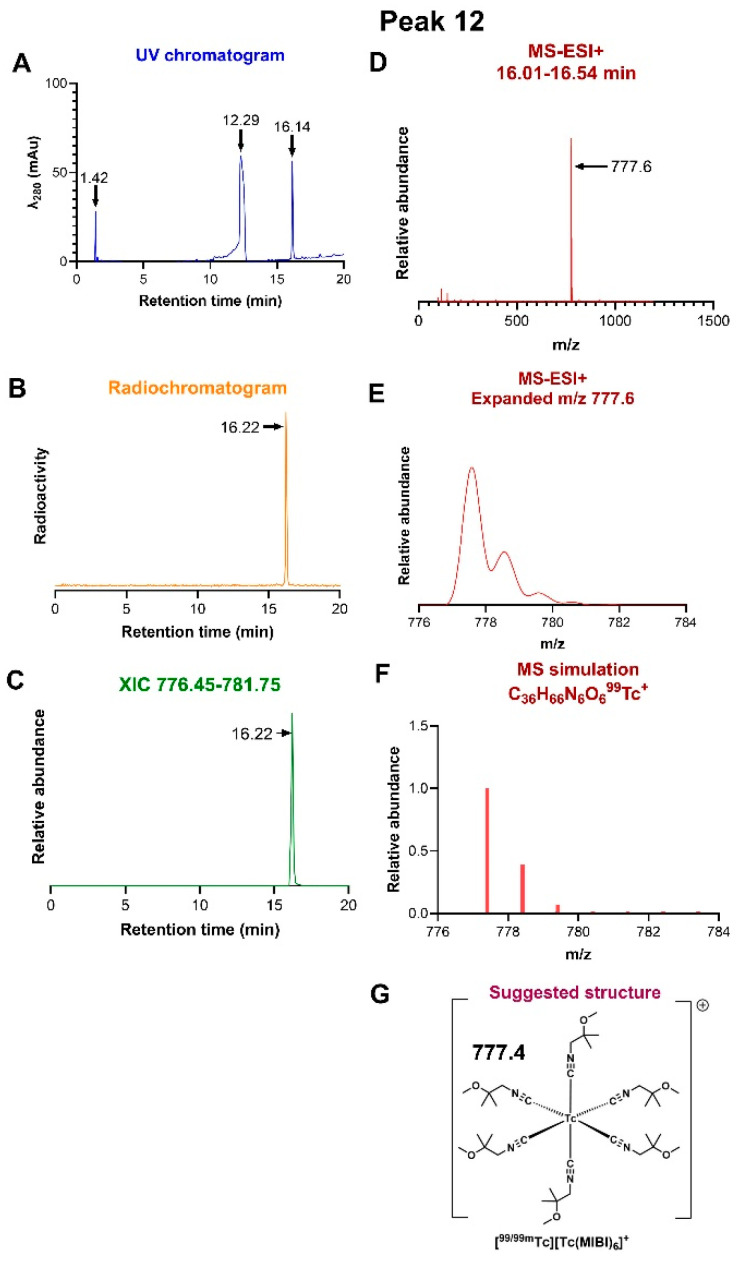
LC-MS of radiochromatogram peak 12, identified as [^99m^Tc][Tc(MIBI)_6_]^+^. (**A**) UV chromatogram showing elution of Cu-MIBI complexes at ca. 12.3 min and [Tc(MIBI)_6_]^+^ at ca. 16.2 min. (**B**) Radiochromatogram, showing a single radioactive peak at 16.2 min. (**C**) Extracted ion chromatogram of ions with *m*/*z* in the range 776.45–781.75, showing that the species with *m*/*z* 777.6 only elutes in the fraction at 16.2 min. (**D**) MS-ESI+ of the radioactive fraction eluting between 16.01 and 16.54 min, across the *m*/*z* range 0–1500. (**E**) expanded view of the ion with *m*/*z* = 777.6 showing ^13^C satellites (**F**) simulation of the assigned ion [Tc(MIBI)_6_]^+^, C_36_H_66_N_6_O_6_Tc^+^. (**G**) Proposed structure of the ion assigned to *m*/*z* 777.6.

**Figure 5:**
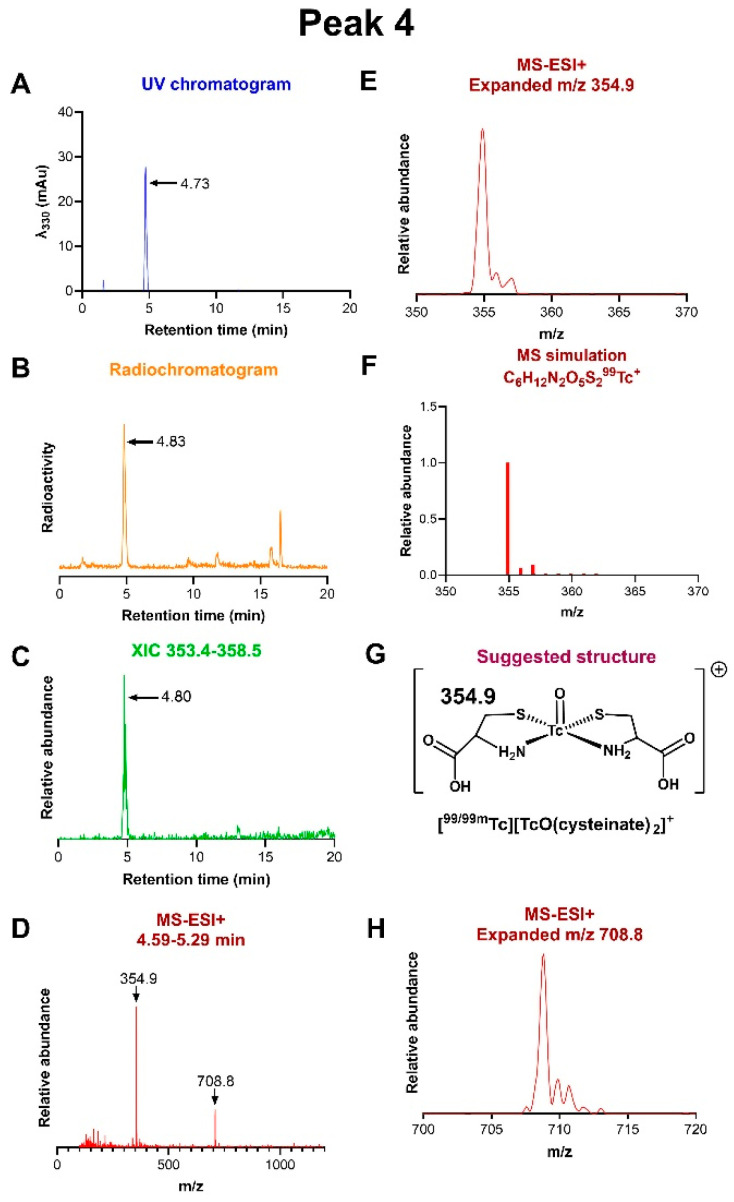
LC-MS of radiochromatogram peak 4. (**A**) UV chromatogram. (**B**) Radiochromatogram, showing a dominant radioactive peak at 4.8 min. (**C**) Extracted ion chromatogram of ions with *m*/*z* in the range 353.4–358.5, showing that the species with *m*/*z* 354.9 only elutes in the fraction at 4.8 min (peak 4). (**D**) MS-ESI+ of the radioactive fraction eluting between 4.59 and 5.29 min, across the *m*/*z* range 0–1500. (**E**) Expanded view of the ion with *m*/*z* = 354.9 showing ^13^C satellites. (**F**) Simulation of the assigned ion [TcO(cysteinate)_2_]^+^, C_6_H_12_N_2_O_5_S_2_Tc^+^. (**G**) Proposed structure of the ion assigned to *m*/*z* 354.9. (**H**) Expanded view of peak with *m*/*z* = 708.8, assigned to a dimer of the structure shown in (**G**).

*Peak 12:* As a standard both to validate the methodology and determine the chemical identity of the final radiopharmaceutical, carrier-added [^99/99m^Tc][^99m^Tc][Tc(MIBI)_6_]^+^ was produced by reacting a Technescan kit (100%) with [^99m^Tc][TcO_4_]^−^ and [NH_4_][^99^TcO_4_] (25 nmol) at 100 °C for 10 min. Figure 4 shows the combined optical-radio-LC-MS-ESI+ (positive ion mode) along with the resulting structural assignment. In this example, the chromatographic fraction examined by MS-ESI+ was defined by the radiochromatogram, which showed a single peak eluting at 16.22 min (Figure 5B), corresponding to a UV peak—Figure 4A—at the same elution time when corrected for flow time between detectors (N.B. this is not a precise match to the elution time reported above for this species because of the different HPLC configurations). The fraction eluting between 16.01 and 16.54 min was selected for MS-ESI+ analysis, thus excluding all species eluting outside these limits. The spectrum showed a single dominant peak, with *m*/*z* = 777.6 (Figure 4D) corresponding to C_36_H_66_N_6_O_6_Tc^+^ (expected 777.4; an expanded view of this peak is shown in Figure 4E and a simulation in Figure 4F), confirming its molecular identity as the Tc(I) complex [Tc(MIBI)_6_]^+^. An extended ion chromatogram (XIC) derived by searching for this ion across the chromatogram, shown in Figure 4C, confirms that this ion was only found to be associated with the radioactive peak at 16.22 min (corresponding to peak 12 in Figure 3).

*Peaks 1–3:* Under reaction conditions that optimise the earliest intermediates (both in the sense of the earliest-formed in the incubation and the earliest-eluting in the chromatogram; peaks 1–3, Figure 3), radioactive fractions eluted between 0 and 4.5 min. All MS-ESI+ peaks detected in this fraction could be readily assigned to kit components (citric acid, mannitol, cysteine, and their derivatives, see Figures S12–S16); additional ions containing technetium were not detected, despite the fractions being radioactive. This indicates that the Tc-containing species represented by peaks 1–3 are poorly ionised or readily fragmented during the ESI+ process used, and so are not detected.

*Peak 4:* The fraction eluting between 4.59 and 5.29 min contained a single radioactive species eluting at 4.83 min (Figure 5, corresponding to peak 4 in Figure 3). Its mass spectrum showed two predominant ions with *m*/*z* = 454.9 and 708.8, corresponding to C_6_H_12_N_2_O_5_S_2_Tc^+^ and C_12_H_23_N_4_O_10_S_4_Tc_2_^+^, respectively, consistent with the Tc(V) oxo complex [TcO(cysteinate)_2_]^+^ (Figure 5) and its dimer. The precise *m*/*z* indicates the number of protons, confirming the oxidation state of technetium as Tc(V) in both cases.

**Figure 6:**
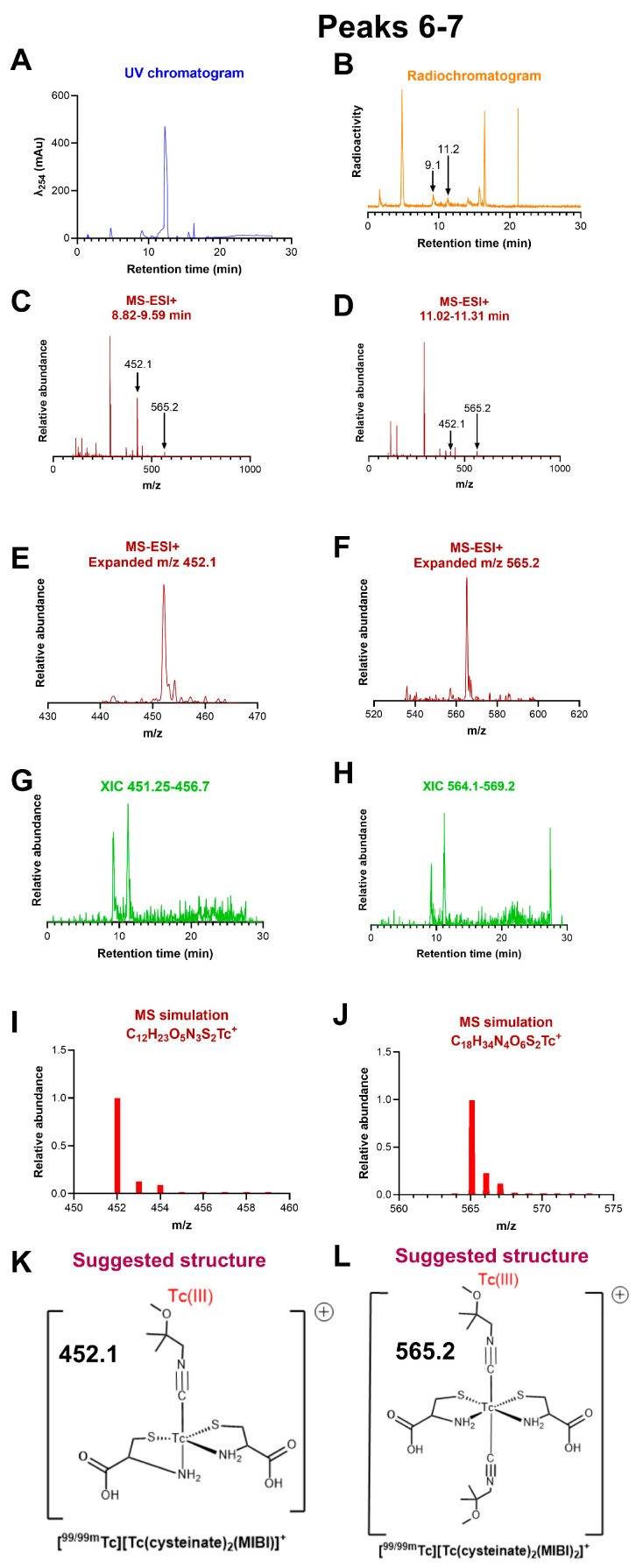
LC-MS of radiochromatogram peaks 6 and 7. (**A**) UV chromatogram. (**B**) Radiochromatogram, showing radioactive peaks analysed by MS at 9.1 and 11.2 min. (**C**) MS-ESI+ of the radioactive fraction eluting between 8.82 and 9.59 min, across the *m*/*z* range 0–1000. (**D**) MS-ESI+ of the radioactive fraction eluting between 11.02 and 11.31 min, across the *m*/*z* range 0–1000. (**E**) Expanded view of the ion with *m*/*z* = 452.1 showing ^13^C satellites. (**F**) Expanded view of the ion with *m*/*z* = 565.2 showing ^13^C satellites. (**G**) Extracted ion chromatogram of ions with *m*/*z* in the range 451.3–456.7, showing that the species with *m*/*z* 452.1 originates both in peaks 6 and 7. (**H**) Extracted ion chromatogram of ions with *m*/*z* in the range 564.1–569.2, showing that the species with *m*/*z* 465.2 originates both in peaks 6 and 7. (**I**) Simulation of the assigned ion [Tc(cysteinate)_2_(MIBI)]^+^, C_12_H_23_N_3_O_5_S_2_Tc^+^. (**J**) Simulation of the assigned ion [Tc(cysteinate)_2_(MIBI)_2_]^+^, C_18_H_34_N_4_O_6_S_2_Tc^+^. (**K**) Proposed structure of the ion assigned to *m*/*z* = 452.1. (**L**) Proposed structure of the ion assigned to *m*/*z* = 565.2.

*Peaks 5–9:* MS-ESI+ analysis of the middle-eluting species with intermediate lipophilicity (represented by peaks 5–9 in Figure 3) showed mainly MS-ESI+ peaks that could be accounted for by non-Tc-containing kit components (Figures S15 and S16) despite being radioactive. Peaks 6 and 7 (Figure 3), however, also showed small peaks with *m*/*z* = 452.1 and 565.2, matching C_12_H_23_O_5_N_3_S_2_Tc^+^ (expected 452.0) and C_18_H_34_O_6_N_4_S_2_Tc^+^ (expected 565.1) (see Figure 6). These are assignable to molecular ions [Tc(cysteinate)_2_(MIBI)]^+^ and [Tc(cysteinate)_2_(MIBI)_2_]^+^, with the number of protons indicating the oxidation state of technetium as Tc(III). Since both molecular ions originated from both peaks 6 and 7, it may be that these peaks represent isomers of [Tc(cysteinate)_2_(MIBI)_2_]^+^, which readily fragment by losing MIBI during the ESI+ ionisation process. Peaks 5, 8, and 9 evidently contain unidentified Tc species that have low abundance in the reaction and ionise poorly or fragment in the ESI+ mode.

**Figure 7:**
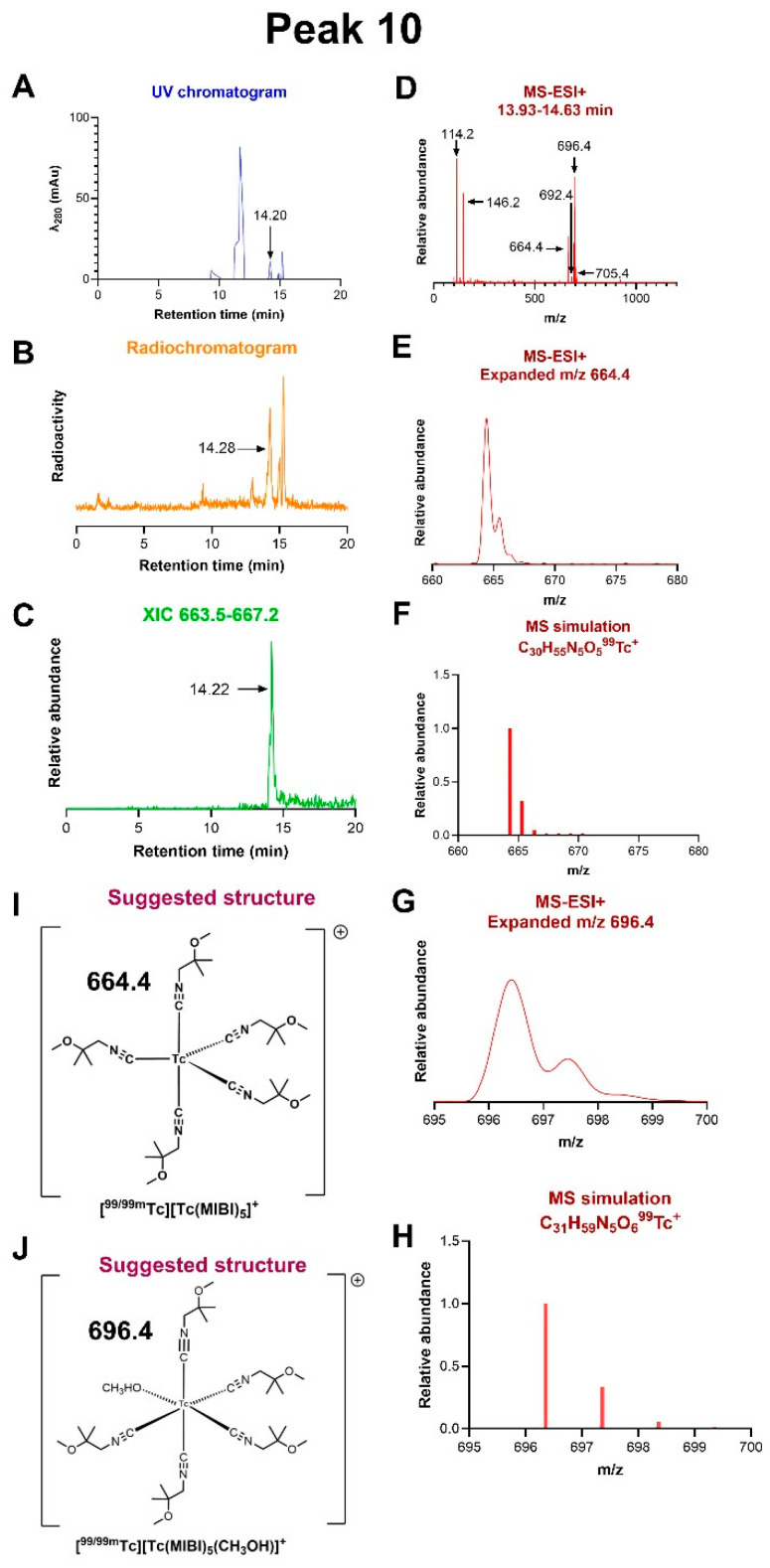
LC-MS of radiochromatogram peak 10. (**A**) UV chromatogram. (**B**) Radiochromatogram, showing a significant radioactive peak at 14.3 min. (**C**) Extracted ion chromatogram of ions with *m*/*z* in the range 663.5–667.2, showing that the species with *m*/*z* 664.4 only elutes in the fraction at 14.2 min. (**D**) MS-ESI+ of the radioactive fraction eluting between 13.93 and 14.63 min, across the *m*/*z* range 0–1500, showing multiple molecular ions, of which those at *m*/*z* 696.4 and 664.4 are dominant. (**E**) Expanded view of the ion with *m*/*z* = 664.4 showing ^13^C satellites. (**F**) Simulation of the assigned ion [Tc(MIBI)_5_]^+^, C_30_H_55_N_5_O_5_Tc^+^. (**G**) Expanded view of the ion with *m*/*z* = 696.4 showing ^13^C satellites. (**H**) Simulation of the assigned ion [Tc(MIBI)_5_(MeOH)]^+^, C_31_H_59_N_5_O_6_Tc^+^. (**I**) Proposed structure of the ion assigned to *m*/*z* 664.4. (**J**) Proposed structure of the ion assigned to *m*/*z* 696.4.

*Peak 10:* The most lipophilic radioactive group of species, eluting between 14 and 18 min in HPLC method 1 (Figure 3), includes the final product [Tc(MIBI)_6_]^+^ (peak 12) and two earlier-eluting peaks (10 and 11). The MS-ESI+ spectrum of peak 10 (Figure 7), capturing the fraction eluting between 13.93 and 14.63 min, gave molecular ions with *m*/*z* = 696.4 (corresponding to C_31_H_59_N_5_O_6_Tc^+^, expected 696.4) and 664.4 (corresponding to C_30_H_55_N_5_O_5_Tc^+^, expected 664.3). These would be accounted for by [Tc(MIBI)_5_(MeOH)]^+^ and [Tc(MIBI)_5_]^+^, respectively, with the number of protons confirming the oxidation state of technetium as Tc(I) in both. Replacing methanol in the HPLC mobile phase with acetonitrile (HPLC method 3) gave a similar radiochromatogram (Figure S17), in which the fraction eluting between 15.97 and 16.49 min (containing a radioactive peak at 16.09 min, assumed to correspond to peak 11 in Figure 3 with HPLC method 1) gave a mass spectrum with a dominant peak at *m*/*z* = 705.0, corresponding to C_32_H_58_N_6_O_5_Tc^+^ (expected 705.3, assigned as [Tc(MIBI)_5_(MeCN)]^+^), and a minor peak at *m*/*z* 664.0, assigned as [Tc(MIBI)_5_]^+^ as discussed above (Figure 7), both containing technetium in oxidation state Tc(I).

**Figure 8:**
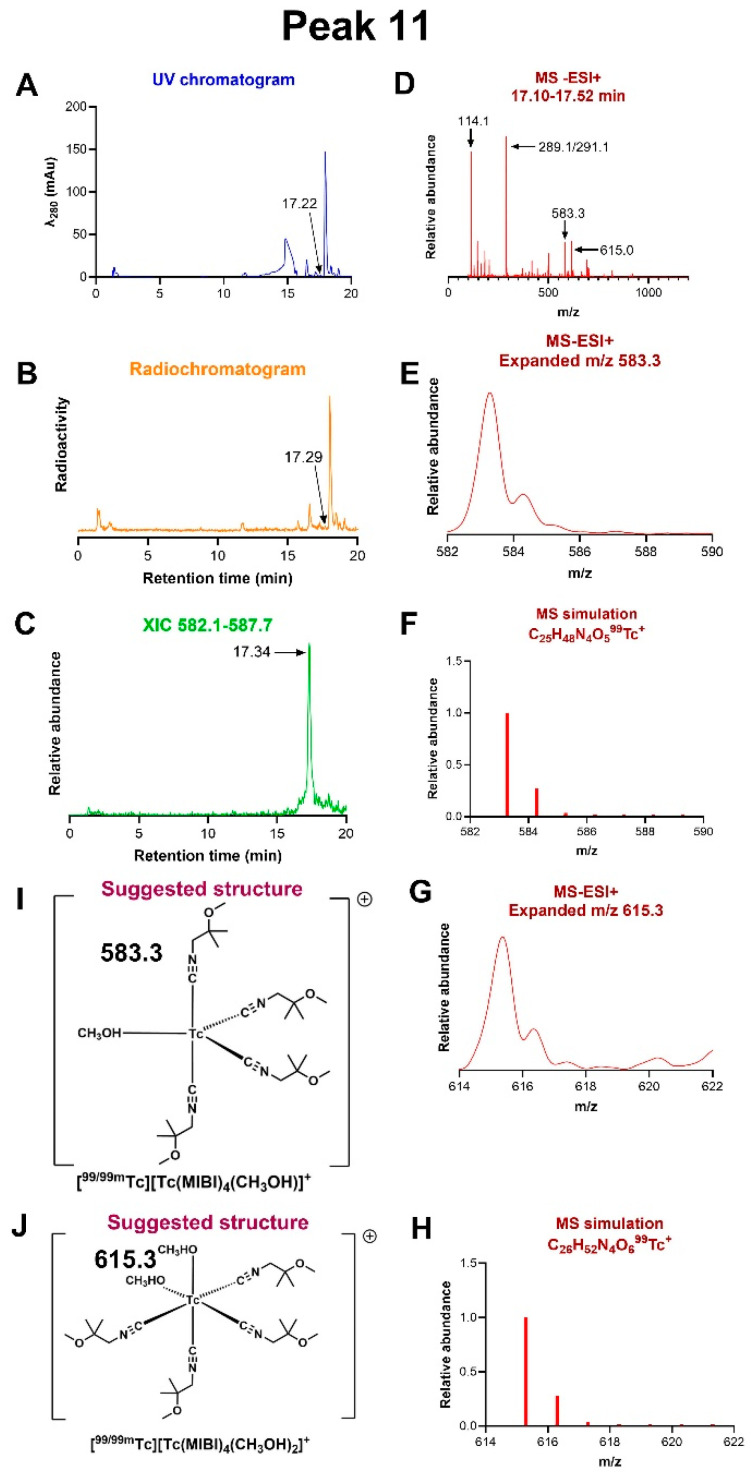
LC-MS of radiochromatogram peak 11. (**A**) UV chromatogram. (**B**) Radiochromatogram, showing radioactive peaks between 15 and 18 min. (**C**) Extracted ion chromatogram of ions with *m*/*z* in the range 582.1–587.7, showing that the species with *m*/*z* 583.3 only elutes in the fraction at 17.3 min. (**D**) MS-ESI+ of the radioactive fraction eluting between 17.10 and 17.52 min, across the *m*/*z* range 0–1500, showing multiple molecular ions, of which those at *m*/*z* 583.3 and 615.3 can be assigned to Tc-containing species. (**E**) Expanded view of the ion with *m*/*z* = 583.3 showing ^13^C satellites. (**F**) Simulation of the assigned ion [Tc(MIBI)_4_(MeOH)]^+^, C_25_H_48_N_4_O_5_Tc^+^. (**G**) Expanded view of the ion with *m*/*z* = 615.3 showing ^13^C satellites. (**H**) Simulation of the assigned ion [Tc(MIBI)_4_(MeOH)_2_]^+^, C_26_H_52_N_4_O_6_Tc^+^. (**I**) Proposed structure of the ion assigned to *m*/*z* = 583.3. (**J**): Proposed structure of the ion assigned to *m*/*z* = 615.3.

*Peak 11:* In analysing peak 11 (Figure 3), to improve its separation from peak 12, the gradient was extended using HPLC method 2, in which peak 11 eluted at 17.3 min. The fraction eluting between 17.1 and 17.5 min was analysed by MS-ESI+, producing molecular ions with *m*/*z* = 615.0 and 583.3 (Figure 8), corresponding to C_26_H_52_N_4_O_6_Tc^+^ (expected 615.3) and C_25_H_48_N_4_O_5_Tc^+^ (expected 583.3). These are assigned as [Tc(MIBI)_4_(MeOH)_2_]^+^ and [Tc(MIBI)_4_(MeOH)]^+^, respectively. A small peak with *m*/*z* ca. 502 was also observed, tentatively assignable to [Tc(MIBI)_3_(MeOH)_2_]^+^, and another with *m*/*z* ca. 696, which was more prominent in peak 10, assignable to [Tc(MIBI)(MeOH)]^+^. This fraction also gave rise to peaks with *m*/*z* = 114.1 and 289.1, assignable to (MIBI+H)^+^ and [Cu(MIBI)_2_]^+^, respectively. Replacing methanol in the mobile phase with acetonitrile (method 3) led to the equivalent peak eluting at 15.2 min, captured within the fraction eluting between 15.0 and 15.4 min (see Supplementary Figure S18). This fraction gave a single major ion with *m*/*z* = 633.0, corresponding to C_28_H_50_N_6_O_4_Tc^+^ (expected 633.3), and a minor ion with *m*/*z* ca. 592, corresponding to C_26_H_47_N_5_O_4_Tc^+^ (expected 592.3). These can be assigned to [Tc(MIBI)_4_(MeCN)_2_]^+^ and [Tc(MIBI)_4_(MeCN)]^+^, respectively, both containing Tc(I).

It is also worth noting that no mass spectra of radioactive fractions contained any ions whose isotope distribution indicated the presence of chlorine or tin. The only ions detected containing copper were attributable to Cu(I) MIBI complexes with no additional moieties present. Electrospray ionisation mass spectrometry in negative mode (MS-ESI-) produced no additional interpretable data.

## 3. Discussion

The reactions taking place upon the reconstitution and heating of the kit vial for the production of [^99m^Tc][Tc(MIBI)_6_]^+^ are tantamount to a compendium of the known chemical characteristics of technetium, spanning as they do the range of oxidation states VII, V, III, and I, and the required range of ligands required to stabilise them—chelating and monodentate, with donor atoms that are hard and soft, pi-donor and acceptor, anionic and uncharged. The aim of the work described here is to begin to unravel the pathway, identify intermediates, and relate them to the known body of characterised technetium chemistry, using a combination of radio-HPLC and MS-ESI+ and modifying conditions (temperature, incubation time) and the kit components to generate insight into the role of individual reagents.

The complexity of the pathway and the variety of technetium chemistry within it are confirmed by the large number of technetium-containing intermediates detected in our radio-HPLC analyses, numbering at least 11 (not including the starting [^99m^Tc][TcO_4_]^−^ and the final product [^99m^Tc][Tc(MIBI)_6_]^+^). This figure almost certainly represents an underestimate, since there will be intermediates that are too short-lived—formed too slowly and consumed too quickly—to be detectable by radio-HPLC. It is clear that each peak represents an intermediate and not a by-product because all disappeared upon heating to 100 °C for 10 min, leaving only [^99m^Tc][Tc(MIBI)_6_]^+^ (peak 12). The relative lipophilicity of the various species, as inferred from reverse-phase radio-HPLC elution times, roughly maps on to the progress of the reaction, with the most hydrophilic species formed early in the pathway and eluting early in the chromatogram, and the most lipophilic formed later and eluting later, with the final product [^99m^Tc][Tc(MIBI)_6_]^+^ eluting last of all.

We were encouraged to accept the relevance of the bespoke kits, with individual components absent or reduced, by the observation that the modifications in them produced no new radio-HPLC peaks; only their relative abundance changed. Radio-HPLC of bespoke kit preparations (Tables S7 and S8; Figures S10 and S11) indicates that the earliest-formed intermediates, represented by peaks 1–3, which do not appear in the absence of citric acid but do appear in the absence of mannitol and cysteine, are technetium citrate complexes. Unfortunately, these peaks did not produce assignable technetium-containing molecular ions in their mass spectra, and so they cannot be definitively identified. The absence of any radio-HPLC peaks not assignable to [^99m^Tc][TcO_4_]^−^ when citric acid and cysteine are absent, suggests that without one or both of these reagents, [^99m^Tc][TcO_4_]^−^ is not reduced; they assist indirectly by generating the acidic conditions required for reduction of [^99m^Tc][TcO_4_]^−^ by stannous chloride.

The time- and temperature-dependencies of the abundance of peaks 1–4 show that the early intermediates represented by peaks 1–3 give rise in turn to peak 4. The latter appears only when cysteine is present, with or without citric acid and mannitol; moreover, when MIBI is absent, the reaction proceeds no further than peak 4. These observations suggest that peak 4 represents a cysteine complex. Its likely structure, suggested in Figure 5, is based on the definitive formula and oxidation state indicated by MS-ESI+ (Figure 5) combined with the body of literature on characterised Tc(V)-mono-oxo complexes with nitrogen and thiolate ligands [26]. In particular, cysteine complexes are known to form when [^99m^Tc][TcO_4_]^−^ is reduced by stannous chloride in the presence of cysteine. Despite extensive UV-visible spectroscopy and chromatographic studies [27,28], their structures have not been definitively identified; however, fully structurally characterised rhenium analogues, with a Re(V)oxo core chelated by two cysteinate ligands [29], are in accord with the structure proposed in Figure 5; at increased pH, one of the pendant carboxylate groups may coordinate trans to the oxo group, as seen in the X-ray crystal structure of the rhenium complex [29], but this cannot be determined from the mass spectra. The possibility of carboxylate coordination trans to the oxo group provides a mechanism by which the dimer seen in the mass spectrum could form, by coordination of a dangling carboxylate of one complex to the technetium of the other.

It is clear from the observations discussed so far that MIBI and its copper complexes play no part in the reactions up to and including formation of the Tc(V) intermediate [TcO(cysteinate)_2_]^+^ (peak 4). Equally clearly, all peaks eluting later (peaks 5–12) do not appear without the presence of MIBI and, given their progressively increased lipophilicity, probably incorporate one or more MIBI into their structure. Unfortunately, MS-ESI+ spectra of peaks 5, 8, and 9 gave no peaks assignable to technetium complexes, despite containing radioactivity. MS-ESI+ spectra from peaks 6 and 7 (Figure 6) gave weak ions attributable to technetium complexes containing both cysteinate and MIBI ligands, with technetium in oxidation state Tc(III) (but none assignable to Tc(V) or Tc(I)). The structures suggested in Figure 6 are based on MS-ESI+ data discussed above in relation to known structures of Tc(III) complexes containing a mix of anionic (including thiolate) and pi-accepting ligands (such as carbonyl, tertiary phosphine, nitrosyl and including isonitriles), including **2**, **3**, and **4** in Figure 2, and rhenium complexes [Re(SC_6_H_3_Pr^i^_2_)_4_(NO)] [30], [Re(SC_6_H_3_Pr^i^_2_)_3_(NCR)_2_], [Re(SC_6_H_3_Pr^i^_2_)_3_(NCR)(CO)], and [Re(SC_6_H_3_Pr^i^_2_)_3_(CO)_2_] (R = Me or Bu^t^) [31]. Technetium conjugates of a cysteine-containing peptide also gave mass spectra consistent with Tc(III) bound by a tertiary phosphine and a mix of thiolato- and amino-ligands [32].

Peaks 10 and 11 represent intermediates that are more lipophilic and appear later in the reaction than peaks 5–9. The MS-ESI spectrum of peak 10 is dominated by an ion identified as [Tc(MIBI)_5_]^+^, with and without methanol as a sixth ligand. The methanol ligand is replaced by acetonitrile when the methanol in the HPLC mobile phase is replaced by acetonitrile (HPLC method 3); since neither is present in the reaction, these ligands must arise from the HPLC mobile phase, indicating that the sixth coordination site in [Tc(MIBI)_5_]^+^ in the pre-HPLC reaction mixture is vacant or filled by a labile ligand that is easily displaced on-column under the HPLC conditions. This labile ligand cannot be MIBI since [Tc(MIBI)_6_]^+^ (peak 12) does not undergo ligand substitution under these conditions. Any potential ligand present in the kit, including water, could fill this role. The MS-ESI+ spectrum of peak 11 is consistent with a core of [Tc(MIBI)_4_]^+^ with one or two methanol ligands—again arising from the HPLC mobile phase and similarly replaced by acetonitrile in the acetonitrile-based mobile phase. Again, we conclude that in the reaction mixture, these two non-MIBI-occupied sites are vacant or filled by another unidentified labile ligand, such as water. The number of protons in the formula identifies the technetium oxidation state in both 10 and 11 as Tc(I). A very small peak in this fraction suggests the possible presence of [Tc(MIBI)_3_]^+^ fragments as well. It is thus clear that at least the fourth, fifth, and sixth MIBI ligands are added to the technetium after its reduction to Tc(I).

The complexity of the reaction revealed by radio-HPLC highlights the gross oversimplification implicit in TLC or iTLC as a quality control criterion for this radiopharmaceutical. The rationale for the selection of these methods (e.g., those in Table S1, including the manufacturers’ recommended method) appears to be to distinguish the final product [^99m^Tc][Tc(MIBI)_6_]^+^ from [^99m^Tc][TcO_4_]^−^. However, HPLC indicates that this is not a logical rationale because [^99m^Tc][TcO_4_]^−^ disappears very quickly from the reaction mixture under all conditions (even at 0 °C) and is therefore not a likely impurity; the many other intermediates are far more likely to be present as impurities, and many of them evidently co-elute with [^99m^Tc][Tc(MIBI)_6_]^+^ on TLC (as evidenced by Figure S23). A combination of TLC (with a reverse-phase stationary phase and an unusually complex mobile phase consisting of tetrahydrofuran, aqueous ammonium acetate, methanol, and acetonitrile) and HPLC (C-18 reverse-phase column with isocratic mobile phase consisting of aqueous ammonium acetate, acetonitrile and methanol) has been suggested as a quality control method in a World Health Organisation monograph [33] but is not in widespread use or recommended by manufacturers. It aims to quantify the radiochemical impurities of pertechnetate, colloidal technetium, and a Tc(I) hexakis isonitrile complex [Tc(MIBI)_5_(1-(isocyano-κC)-2-methylprop-1-ene)]+ (an impurity not detected in our experiments), but none of the other intermediates identified in our experiments. Reasons for choosing to detect the latter impurity, or the proposed mechanism of its formation, are not given. As acknowledged in the monograph, HPLC alone is not a suitable quality control method because it cannot quantify any radioactivity that does not elute from the column (which can be a significant fraction as seen in our experiments), and in any case is too costly and cumbersome for routine use. TLC/iTLC is unlikely to offer the resolution required to detect all potential impurities individually. An alternative and practical approach may be to identify one intermediate/impurity or a group of them to serve as a reliable predictor of satisfactory yield, that is, one which is always present when the yield of [^99m^Tc][Tc(MIBI)_6_]^+^ is unsatisfactory. Our experiments (Figure 3 and Figures S4–S10) suggest that the intermediates appearing as peaks 10 and 11 (Figure 3) might fulfil this purpose; since they have similar elution time on reverse-phase HPLC, they are unlikely to be distinguishable by TLC/iTLC, but the lability towards substitution of the aqua, methanol, or acetonitrile ligands may offer a way, subject to future investigation, to selectively trap them on a stationary phase with an immobilisedligand such as imidazole or pyridine.

## 4. Materials and Methods

[^99m^Tc][TcO_4_]^−^ was eluted daily in saline (Ultra-TechneKow FM Eluent, Curium, Westerduinweg, The Netherlands) from a ^99^Mo/^99m^Tc generator (Mallinckrodt Pharmaceuticals, Petten, The Netherlands) and added to Technescan MIBI kits (Mallinckrodt Pharmaceuticals, Petten, The Netherlands) or to bespoke in-house kits with individual components reduced or absent (see Tables S7 and S8) prepared using [Cu(MIBI)_4_][BF_4_] (Merck KGaA, Darmstadt, Germany), L-cysteine hydrochloride monohydrate (Alfa Aesar, Thermo Scientific Chemicals, Waltham, MA, USA), D-mannitol (Sigma-Aldrich, Merck KGaA, Darmstadt, Germany), sodium citrate dihydrate (Sigma-Aldrich, Merck KGaA, Darmstadt, Germany), SnCl_2_·2H_2_O (Alfa Aesar, Thermo Fisher Scientific, Waltham, MA, USA), and HCl (Sigma-Aldrich, Merck KGaA, Darmstadt, Germany). The pH of solutions was determined with pH strips 0–14 (Fisherbrand, Thermo Fisher Scientific, Waltham, MA, USA) and 7.0–14.0 (Sigma Chemical Company, St. Louis, MO, USA). Vials were incubated in an ice bath, at room temperature, or at 100 °C.

*Radio-TLC* analysis was performed using two methods (Table S2). Method 1 (recommended by the kit manufacturer) comprised aluminium oxide/ethanol. Method 2 (also used for the clinical release of the radiopharmaceutical) comprised two strips: A, comprising ITLC-SG/saline, and B, comprising Whatman paper/butanone. Strips were scanned with a LabLogic (Sheffield, UK) Flow-Count radio-TLC scanner and analysed with Laura software (version 6.2.6.22 SP1).

*Radio-HPLC* analysis was performed with three systems: 1: Agilent technologies 1200 series, with UV detection at 254 nm and a Raytest GABI star radioactivity detector, controlled and analysed with GINA Start 5.8 (Elysia-Raytest GmbH, Straubenhardt, Germany); 2: Agilent 1260 Infinity II system with UV/visible detection at 220, 254, 280, or 330 nm and dual scan-RAM gamma detection (Agilent Technologies, Inc. (Waldbronn, Germany)), controlled and analysed with Laura software (version 6.2.6.22 SP1); 3: Agilent 1260 Infinity system with gamma detector (B-FC-3200), UV detection at 220, 254, 280, or 330 nm, and mass spectrometer (see below) analysed with Advion Data Express (version 5.1.0.2) and Advion Mass Express (version 5.1.0.2). A reversed-phase C18 column (Eclipse XDB C18, 5 μm, 4.6 mm × 150 mm), referred to throughout as “C18”, was used with each system. Size-exclusion HPLC was performed as described in the Supplementary Information. Elution gradients, comprising water–methanol or water–acetonitrile as the mobile phase, each with a formic acid modifier, are detailed in Supplementary Table S2. Radioactivity retained on the HPLC column was estimated as the difference between the total injected activity and the activity of all fractions collected in a 50 mL Falcon tube after the total duration of the HPLC run, as measured in the dose calibrator. Data were imported into GraphPad Prism (version 10.6.1.892) for presentation.

*Reaction conditions to detect maximum numbers of [*^99^*^m^Tc]Tc-intermediates* were determined by varying temperature, time, concentration of kit constituents (collectively and individually, including excluding individual components), adding carrier [^99^Tc]pertechnetate, and by fractionating Technescan MIBI kits and preparing bespoke kits from their individual components, as described in the Supplementary Information. Isolation of individual intermediates by HPLC, and their subsequent re-analysis by HPLC and MS-ESI+, are described in the Supplementary Information.

*Mass spectrometry (MS)* was performed on an Advion Expression Compact MS (Advion Interchim Scientific (formerly Advion, Inc.), Ithaca, NY, USA), free-standing or in-line with HPLC as the third detector (in series with UV as the first and gamma as the second), producing a set of three superimposed chromatograms, enabling technetium-containing peaks to be identified in the radiochromatogram for MS analysis, in low-resolution positive electrospray ionisation mode (ESI+) in the mass-to-charge (*m*/*z*) range of 100–1500, with a scan time of 917 ms and capillary temperature of 250 °C. Negative mode was also attempted, but provided no additional information. To evaluate the potential for fragmentation, high-fragmentation (HF, 40 V) and low-fragmentation (LF, 0 V) source voltages were used. Mass spectrum simulations were performed using Protpi (Version 2.2.29.152, https://www.protpi.ch/Calculator/MassSpecSimulator (accessed on 20 December 2025)). The results of the foregoing experiments were used to identify sets of conditions to optimise the formation of selected radioactive peaks in the radiochromatograms. A summary of the chosen reaction conditions (which included the addition of carrier [^99^Tc]pertechnetate) and their “target” peaks is provided in Table S9. Dilutions with both saline and water were used; the latter modification, intended to help identify any intermediates containing coordinated chloride and to minimise suppression of ionisation of analytes due to high ionic strength, produced no changes in the radiochromatograms. Technetium-free kits and fully formed [^99m^Tc][Tc(MIBI)_6_]^+^, produced by heating the reaction solution at 100 °C for 10 min, were analysed similarly for comparison. Some reaction mixtures were also analysed by MS-ESI+ directly, without radiochromatography, to eliminate the possibility of on-column reactions (which were shown to occur, see Section 2) and facilitate a faster kinetic sampling of the solutions at early time points; no additional information emerged from this approach.

## 5. Conclusions

We have established that in the sestamibi kit, at least 11 intermediates occur en route to [Tc(MIBI)_6_]^+^ that are sufficiently long-lived to be detectable by radio-HPLC. Some of them could be identified by MS-ESI+ and assigned ligand sets and oxidation states. All the species explicitly identified here are plausible in light of the published literature on the nature of technetium complexes in oxidation states (I) to (VII). The reaction proceeds via the formation of one or more Tc(VII)- and/or Tc(V)-citrate complexes, to a relatively long-lived Tc(V) species [TcO(cysteinate)_2_]^+^, which was unequivocally identified by mass spectrometry; until this point, MIBI plays no part. Subsequently, the Tc(V) complex is reduced to the Tc(III) species that retains the two cysteinate ligands but loses the oxo-ligand, which is replaced by one or more MIBI ligands. It remains unclear whether a MIBI ligand is added before this reduction; however, we note that there is evidence in the MS-ESI+ data (dimerisation of [TcO(cysteinate)_2_]^+^) and in the literature [29] that additional ligand binding trans to the oxo-ligand of [TcO(cysteinate)_2_]^+^ can occur, providing an opportunity for MIBI to bind at this point; however, no molecular ion corresponding to a Tc(V) complex with both cysteinate and MIBI ligands was detected. The Tc(III) complex goes on to lose both cysteine ligands and gain MIBI ligands, via hitherto unidentified intermediates possibly represented by radio-HPLC peaks 5, 8, and 9, accompanied by reduction to Tc(I). At least the fifth and sixth MIBI ligands join the complex by displacing more labile ligands after the reduction to Tc(I), to yield the final complex [Tc(MIBI)_6_]^+^, which is relatively unreactive. This sequence of reactions is consistent with the expectation that the pi-donor, predominantly anionic ligands (oxo-anion, cysteine thiolate) are involved in stabilising the higher oxidation state technetium complexes (Tc(V)) while the pi-acceptor, uncharged ligands (isonitrile) are involved in stabilising the lower oxidation state technetium complexes (Tc(I)). The intermediate oxidation state (Tc(III)) is represented by complexes containing a mix of anionic/pi-donor ligands and uncharged pi-acceptor ligands. The complexity evident from radio-HPLC also indicates that the present TLC/iTLC quality control methods and criteria [34], accepted clinically for almost four decades, have no rational scientific basis and should be revisited.

Insights from this study suggest possible technetium coordination chemistry that could contribute to the synthesis of technetium bioconjugates. A possible example is a Tc(III)-isonitrile-cysteinate derivative in which the cysteine is part of a peptide or protein, analogous to the Tc(III)-tertiary phosphine moiety previously shown to bind to a cysteine-containing peptide [32]. Another pursues the analogy of the [Tc(isonitrile)*_n_*]^+^ (*n* = 3, 4, or 5), with the [Tc(CO)_3_]^+^ fragment used for site-specific labelling of polyhistidine tags in proteins [35], offering a possible alternative synthon that can be synthesised under milder conditions. The next step towards deepening the understanding of the intermediates and mechanisms of their interconversion would be to return to the inorganic synthesis of individual intermediates, using bulk amounts of technetium-99, and characterise them with conventional analytical techniques such as NMR, X-ray crystallography, and cyclic voltammetry.

## Data Availability

The original contributions presented in this study are included in the article/Supplementary Materials. Further inquiries can be directed to the corresponding author.

## Supplementary Materials

The following supporting information can be downloaded at https://www.mdpi.com/article/10.3390/molecules31040596/s1: Table S1: TLC methods; Table S2: HPLC methods; Table S3: Summary of reaction conditions used with Technescan MIBI kits; Table S4: Kit fractionation; Table S5: Effect of kit fractionation on percentage of recovered activity by HPLC; Table S6: Reaction conditions used to determine the influence of carrier ^99^Tc; Table S7: Composition of bespoke MIBI kits containing variable amounts of [Cu(MIBI)_4_][BF_4_]; Table S8: Composition of bespoke MIBI kits containing variable amounts of other components; Figure S1: TLC results (method 1); Figure S2: TLC results (method 2); Figure S3: HPLC radiochromatograms of [^99m^Tc][TcO_4_]^−^ and [^99m^Tc][Tc(MIBI)_6_]^+^; Figure S4: Incubation time-dependence of HPLC after room temperature reaction; Figure S5: Incubation time-dependence of HPLC after reaction at 0 °C; Figure S6: Size-exclusion (SEC)-HPLC radiochromatograms; Figure S7: HPLC radiochromatograms of fractionated kits after 5 min room temperature incubation; Figure S8: HPLC radiochromatograms after addition of carrier ^99^Tc, room temperature, 25% of kit; Figure S9: HPLC radiochromatograms after addition of carrier ^99^Tc, room temperature, 45% of kit; Figure S10: HPLC of bespoke kits 1–4, room temperature, as a function of incubation time; Figure S11: HPLC of bespoke kits 5–8, room temperature, as a function of time; Figure S12: LCMS of mannitol; Figure S13: LCMS of citric acid; Figure S14: LCMS of cysteine; Figure S15: LCMS of [Cu(MIBI)_4_][BF_4_], methanol-based mobile phase; Figure S16: LCMS of [Cu(MIBI)_4_][BF_4_], acetonitrile-based mobile phase; Figure S17: LCMS of peak 10, acetonitrile-based mobile phase; Figure S18: LCMS of peak 11, acetonitrile-based mobile phase; Figure S19: HPLC purification and re-analysis of peak 4; Figure S20: HPLC purification and re-analysis of peak 10; Figure S21: HPLC purification and re-analysis of peak 11; Figure S22: HPLC purification and re-analysis of peak 12; Figure S23: Exemplar TLC and HPLC of Technescan MIBI kit incubated on ice for 60 min.

## Abbreviations

The following abbreviations are used in this manuscript:

ESI	Electrospray ionisation
FDA	Food and Drug Administration
HF	High fragmentation
HPLC	High-performance liquid chromatography
LF	Low fragmentation
MIBI	Methoxybutylisonitrile
MS	Mass spectrometry
SPECT	Single-photon emission computed tomography
TLC	Thin-layer chromatography
UV	Ultraviolet
