# Supplementary material for: From [99mTc]pertechnetate to [99mTc]sestamibi: Dissection of a Complex Reaction Sequence Using Radio-LC-MS
Source: Molecules. 2026 Feb 9;31(4):596. doi: 10.3390/molecules31040596 (PMC12943784; doi:10.3390/molecules31040596)
Supplement: Supplementary file 1 [file molecules-31-00596-s001.zip › molecules-4100853-supplementary.pdf]

# From [$^{99m}$Tc]pertechnetate to [$^{99m}$Tc]sestamibi: Dissection of a Complex Reaction Sequence Using Radio-LC-MS

Joana do Mar Ferreira Machado [1], Antonio Shegani [1], Ingebjørg N. Hungnes [1], Truc T. Pham [1], Amaia Carrascal-Miniño [1], Margaret S. Cooper [1], Victoria Gibson [1,2], Levente K. Meszaros [3], Michelle T. Ma [1] and Philip J. Blower [1]*

[1] King's College London, School of Biomedical Engineering and Imaging Sciences, 4th Floor Lambeth Wing, St Thomas' Hospital. London SE1 7EH, London, UK; joana_do_mar.machado@kcl.ac.uk (J.d.M.F.M.); antonio.shegani@kcl.ac.uk (A.S.); ingebjorg.hungnes@kcl.ac.uk (I.H.); truc.pham@kcl.ac.uk (T.T.P.); amaia.minino@kcl.ac.uk (A.C.-M.); margaret.cooper@kcl.ac.uk (M.S.C.); victoria.1.gibson@kcl.ac.uk (V.G.); michelle.ma@kcl.ac.uk (M.T.M.)
[2] Department of Nuclear Medicine, Guy's and St Thomas' NHS Foundation Trust, London, SE1 9RT, UK
[3] Nanomab Technology (UK) Limited, Elstree, Hertfordshire, WD6 3SY, UK; leventemesza-ros@nano-mab.com
*Correspondence: philip.blower@kcl.ac.uk

## Supplementary Information

### TLC methods

In method 1, the radiolabelled mixture (3 µL) was applied at 2 cm from the bottom of the aluminium oxide strip (Bakerflex 1 B-F, J.T. Baker Chemical Co, Phillipsburg, pre-cut strips 2.5 cm x 7.5 cm), and allowed to dry. The strip was placed in ethanol and allowed to develop until it reached 0.5 cm from the top. Then, the strip was removed, dried in air, scanned with a radio-TLC scanner (Flow-count, Lablogic) and analysed with Laura software (version 6.2.6.22 SP1). In method 2, two systems were used (Table S1). In system A, the solution was spotted at 1.5 cm from the bottom of an ITLC-SG strip 1 x 9 cm (Agilent Technologies, Folsom, CA). The strip was placed in saline and allowed to develop until it reached 1 cm from the top. In system B, the solution was spotted at 1.5 cm from the bottom of a Whatman paper strip 1 x 9 cm and allowed to develop in butanone until it reached 1 cm from the top. The strips were scanned with a radio-TLC scanner and analysed with Laura software (as above) (Figs. S1, S2).

***Table S1*** *TLC methods used for the evaluation of reaction products of [$^{99m}$Tc][$TcO_4$]$^-$ with the Technescan MIBI kit.*

| Method | | Mobile phase | Stationary phase | $R_f$ = 0.0-0.1 (origin) | $R_f$ = 1.0 (front) |
|---|---|---|---|---|---|
| 1 | | Ethanol | Aluminium oxide strips | [$^{99m}$Tc]Tc-RH | [$^{99m}$Tc][Tc(MIBI)$_6$]$^+$ |
| 2 | A | Saline | iTLC-SG | [$^{99m}$Tc][Tc(MIBI)$_6$]$^+$ | [$^{99m}$Tc][$TcO_4$]$^-$ and other hydrophilic impurities |
| | B | Butanone | Whatman paper | [$^{99m}$Tc]Tc-RH | [$^{99m}$Tc][$TcO_4$]$^-$ and [$^{99m}$Tc][Tc(MIBI)$_6$]$^+$ |

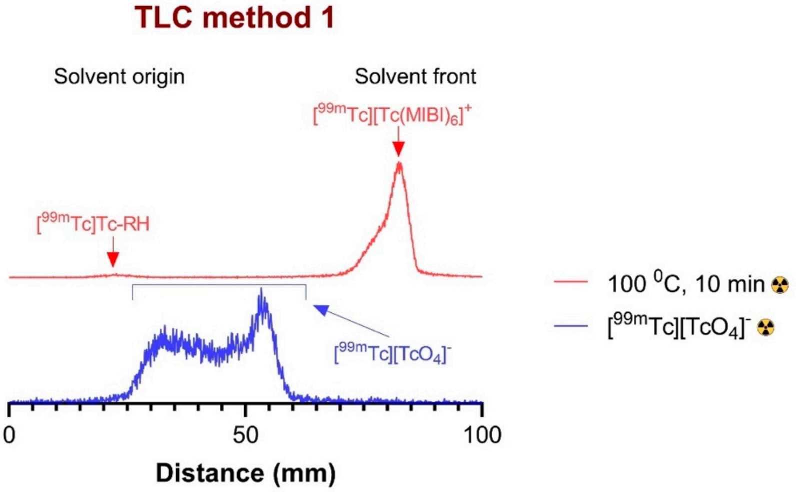


**Figure S 1** *TLC results (method 1, see Table S1) of the solution of [$^{99m}$Tc][TcO$_4$]$^-$ (blue), and the reconstituted kit solution prepared at 100 °C for 10 min (red), showing that [$^{99m}$Tc][Tc(MIBI)$_6$]$^+$ was formed in high radiochemical yield (97.8%) with Rf = 0.824. There was almost negligible formation of [$^{99m}$Tc]Tc-RH, and no [$^{99m}$Tc][TcO$_4$]$^-$.*

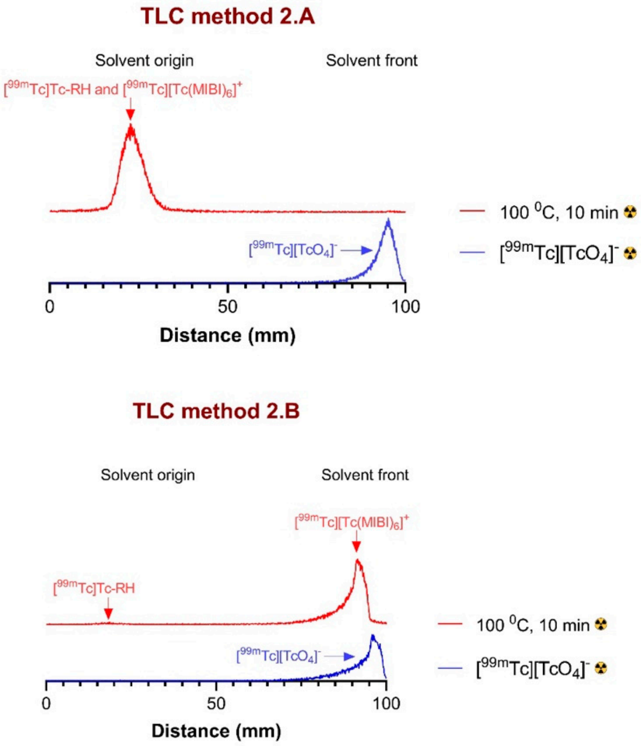


***Figure S 2*** *TLC results (method 2.A (top) and method 2.B (bottom), see Table S1) of [$^{99m}$Tc][TcO$_4$]$^-$, and the reconstituted kit solution prepared at 100 °C for 10 min are represented in blue and red, respectively. In method 2.A, [$^{99m}$Tc][Tc(MIBI)$_6$]$^+$ stayed at the origin together with [$^{99m}$Tc]Tc-RH (Rf = 0.23) indicating a radiochemical yield of >99.0%. In method 2.B, [$^{99m}$Tc][Tc(MIBI)$_6$]$^+$ migrated with the solvent front together with [$^{99m}$Tc][TcO$_4$]$^-$ (Rf = 0.92) indicating a radiochemical yield of 98.0%.*

***Table S 2*** *HPLC methods used to analyse the solution produced by the reaction of [$^{99m}$Tc][$TcO_4$]$^-$ with the Technescan MIBI kit.*

| Method number | Mobile Phase | Gradient system | Column | Flow (mL/min) |
|---|---|---|---|---|
| 1 | **A:** $H_2O$/0.1% FA<br>**B:** $CH_3OH$/0.1% FA | 0-100% B over 20 min | $C_{18}$ | 1 |
| 2 | **A:** $H_2O$/0.1% FA<br>**B:** $CH_3OH$/0.1% FA | 0-50% B, 0-15 min<br>50% B, 15-25 min<br>50-100% B, 25-40 min | $C_{18}$ | 1 |
| 3 | **A:** $H_2O$/0.1% FA<br>**B:** $CH_3CN$/0.1% FA | 0-100% B over 20 min | $C_{18}$ | 1 |
| 4 | **A:** $H_2O$/0.1% FA<br>**B:** $CH_3OH$/0.1% FA | Isocratic, 55% A, 45% B | SEC | 1 |
| 5 | **A:** $H_2O$/0.1% FA<br>**B:** $CH_3CN$/0.1% FA | 0-50% B, 0-15 min<br>50% B, 15-25 min<br>50-100% B, 25-40 min | $C_{18}$ | 1 |
| 6 | **A:** $H_2O$/0.1% FA<br>**B:** $CH_3OH$/0.1% FA | 0% B, isocratic, 3 min | $C_{18}$ | 1 |

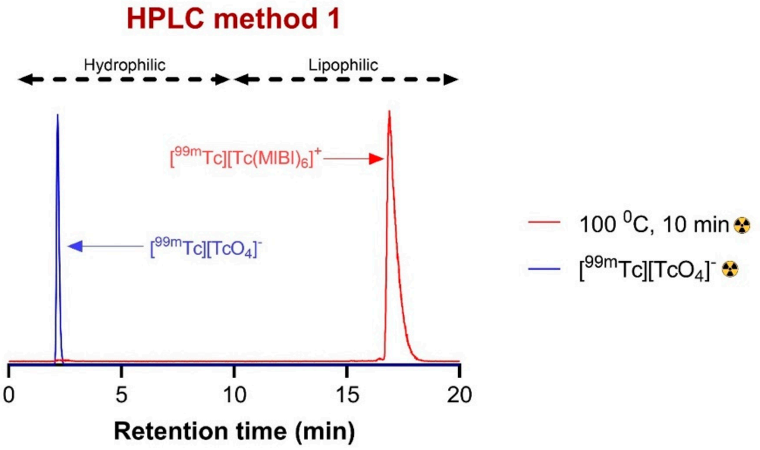


***Figure S 3*** *HPLC radiochromatograms (method 1, see Table S2) of [$^{99m}$Tc][$TcO_4$]$^-$ and [$^{99m}$Tc][Tc(MIBI)$_6$]$^+$ produced with a kit prepared at 100 °C for 10 min (red), and the control [$^{99m}$Tc][$TcO_4$]$^-$ (blue). The red chromatogram shows a major peak that corresponded to putative [$^{99m}$Tc][Tc(MIBI)$_6$]$^+$, with a radiochemical yield of 99.3 %, eluted at 16.9 min.*

***Table S 3*** *Summary of the reaction conditions used with Technescan MIBI kits to determine the influence of temperature and time on the profile of [$^{99m}$Tc]Tc-intermediates.*

| Temperature | Incubation time (min) |
|---|---|
| RT | 0.5 |
| | 1.5 |
| | 3 |
| | 5 |
| | 30 |
| | 60 |
| 100 °C | 10 |
| 0 °C | 0.5 |
| | 1.5 |
| | 3 |
| | 5 |
| | 30 |
| | 60 |

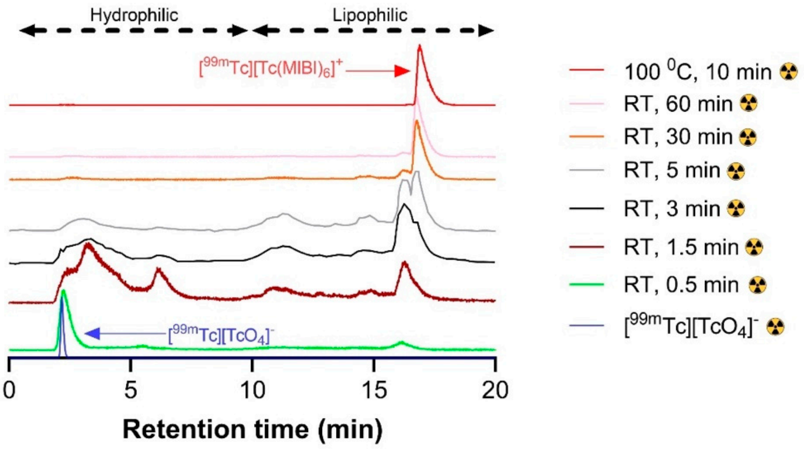


***Figure S 4 .*** *Incubation time dependence of HPLC (method 1, Table S2) after room temperature reaction of $[^{99m}Tc][TcO_4]^-$ with a Technescan MIBI for up to 60 min (Table S3), control prepared at 100 °C (red), and $[^{99m}Tc][TcO_4]^-$ (blue). Reactions performed at RT showed increased formation of $[^{99m}Tc][Tc(MIBI)_6]^+$ as incubation time increased. The peak that co-eluted with $[^{99m}Tc][TcO_4]^-$ almost disappeared after 30 min.*

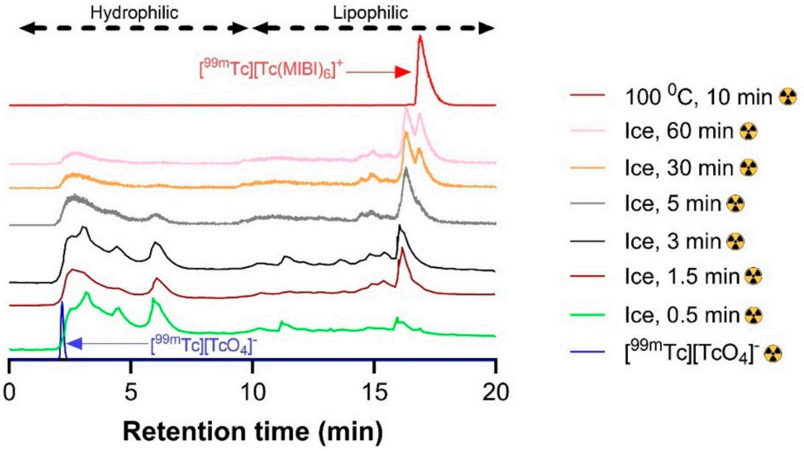


***Figure S 5*** *HPLC radiochromatograms (method 1, Table S2) of the reactions of $[^{99m}Tc][TcO_4]^-$ with a Technescan MIBI kit incubated at 0 °C as a function of time, control $[^{99m}Tc][TcO_4]^-$ (blue) and the reaction performed at 100 °C (red). Performing the reaction at 0 °C significantly delayed the formation of $[^{99m}Tc][Tc(MIBI)_6]^+$.*

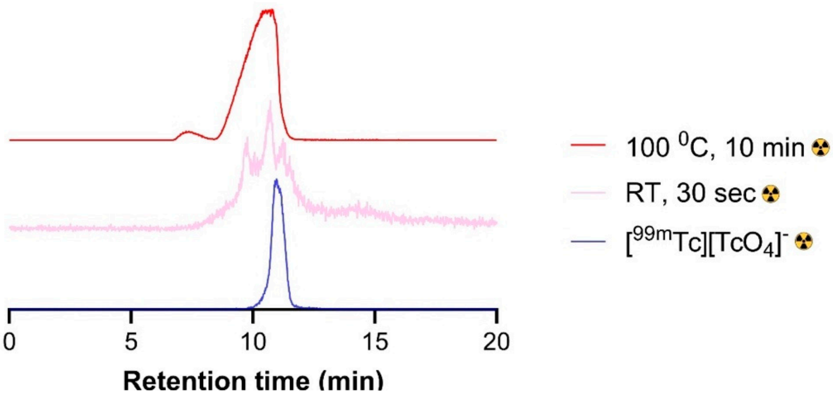


***Figure S 6*** *Size-exclusion (SEC)-HPLC radiochromatograms (method 4, Table S2) following the reaction of $[^{99m}Tc][TcO_4]^-$ with the kit at RT (30 s incubation time) (middle, pink), and controls (reaction prepared at 100 °C (top, red) and $[^{99m}Tc][TcO_4]^-$ (bottom, blue)). The reaction performed at RT showed a broad peak consisting of three overlapping peaks eluted after 7.5 min, and a smaller peak eluting at 14.3 min.*

***Table S 4*** *Kit fractionation used for the radio-LCMS analysis.*

| **Reaction number** | **% of kit components** | **$^{99}$Tc (nmol)** | **Solvent in which the kit is dissolved (saline, $H_2O$)** | **Incubation time (min)** | **Reaction temperature** | **Purpose** |
|---|---|---|---|---|---|---|
| 1 | 0 | 50 | ---------- | ---------- | RT | To identify $[^{99/99m}Tc][TcO_4]^-$ |
| 2 | 100 | 25 | $H_2O$ | 10 | 100 °C | To confirm the identity and elution time of $[^{99/99m}Tc][Tc(MIBI)_6]^+$ |
| 3 | 12.5 | 50 | $H_2O$ | 5 | 0 °C | To characterise early $[^{99/99m}Tc]$Tc-intermediates ($t_R$ = 1.75-2.21 min and $t_R$ = 2.35-3.61 min) |
| 4 | 12.5 | 50 | $H_2O$ | 5 | RT | To characterise middle $[^{99/99m}Tc]$Tc-intermediates ($t_R$ = 4.62-5.29 min) |
| 5 | 45 | 50 | $H_2O$ | 1 | RT | To characterise late $[^{99/99m}Tc]$Tc-intermediates ($t_R$ = 12.81-13.20 min, $t_R$ = 13.86-14.67 min and $t_R$ = 14.81-15.23 min) |
| 6 | 45 | 25 | $H_2O$ | 1 | RT | To investigate the influence of chloride on the profile of $[^{99/99m}Tc]$Tc-intermediates |
| 7 | 45 | 25 | $H_2O$ | 0.5 | 0 °C | To investigate the influence of MS parameters (source voltage span) in the profile of $[^{99/99m}Tc]$Tc-intermediates |
| 8 | 45 | 25 | $H_2O$ | 1 | RT | To investigate the influence of LC solvent system and gradient on the profile of $[^{99/99m}Tc]$Tc-intermediates |
| 9 | 45 | 50 | $H_2O$ | 1 | RT | To characterise purified $[^{99/99m}Tc]$Tc-intermediate-12 after freeze-drying |
| 10 | 12.5 | 50 | $H_2O$ | 1 | RT | To characterise purified $[^{99/99m}Tc]$Tc-intermediate-4 from section 3.3.2 after freeze-drying |
| 11 | 45 | 25 | $H_2O$ | 1 | RT | To investigate whether loading the samples without HPLC column allows the identification of $[^{99/99m}Tc]$-intermediates that do not contain adducts of the LC solvent system |

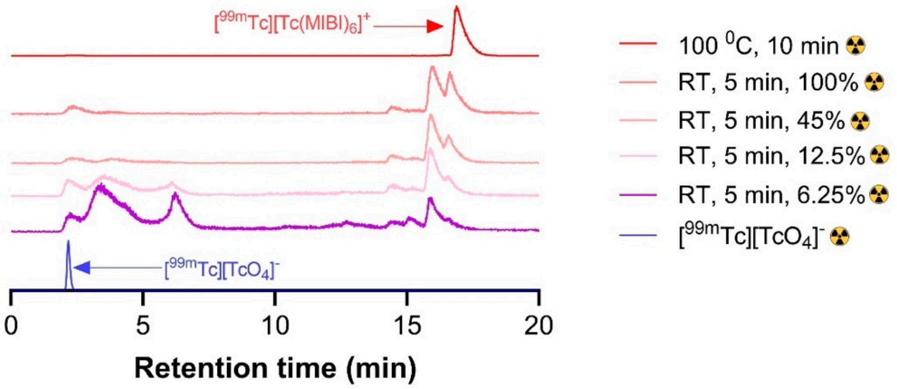


**Figure S 7** *HPLC radiochromatograms (method 1, Table S2) of fractionated kits radiolabelled with $[^{99m}Tc][TcO_4]^-$ at RT for 5 min, control $[^{99m}Tc][TcO_4]^-$ (blue) and the reaction performed at 100 °C (red). Performing the reactions with 6.25% and 12.5% of kit enhanced the formation of the $[^{99m}Tc]$Tc-intermediates eluted between 1.8 min and 7 min. The formation of more lipophilic $[^{99m}Tc]$Tc-intermediates eluting after 12 min was enhanced by higher kit concentrations (45% and 100% of kit components).*

***Table S 5*** *Effect of fractionation on percentage of recovered radioactivity by HPLC.*

| % of kit labelled | Column | Temperature | % recovered |
|---|---|---|---|
| 100 | $C_{18}$ | 100 ºC | 80 |
| 100 | SEC | 100 ºC | 84 |
| 45 | $C_{18}$ | RT | 66.66 |
| 0.0024 | $C_{18}$ | RT | 14.5 |

***Table S 6*** *Reaction conditions used to determine the influence of carrier $^{99}$Tc concentration on the profile of [$^{99m}$Tc]Tc-intermediates.*

| Reaction | Quantity of kit constituents (%) | Temperature | Incubation time (min) | nmol |
|---|---|---|---|---|
| | | | | $^{99}$Tc |
| 1 | 100 | 100 °C | 10 | 25 |
| 2 | 12.5 | RT | 5 | 0 |
| 3 | 12.5 | RT | 5 | 50 |
| 4 | 12.5 | RT | 30 | 50 |
| 5 | 12.5 | RT | 60 | 50 |
| 6 | 12.5 | RT | 120 | 50 |
| 7 | 12.5 | RT | 1440 | 50 |
| 8 | 12.5 | RT | 5 | 100 |
| 9 | 12.5 | RT | 5 | 25 |
| 10 | 45 | RT | 5 | 0 |
| 11 | 45 | RT | 0.5 | 50 |
| 12 | 45 | RT | 1 | 50 |
| 13 | 45 | RT | 3 | 50 |
| 14 | 45 | RT | 5 | 50 |
| 15 | 45 | RT | 10 | 50 |
| 16 | 45 | RT | 30 | 50 |
| 17 | 45 | RT | 60 | 50 |
| 18 | 45 | RT | 120 | 50 |
| 19 | 45 | RT | 5 | 100 |
| 20 | 45 | RT | 5 | 25 |
| 21 | 12.5 | 0 °C | 5 | 0 |
| 22 | 45 | 0 °C | 5 | 0 |
| 23 | 45 | 0 °C | 5 | 100 |
| 24 | 45 | 0 °C | 5 | 50 |
| 25 | 45 | 0 °C | 0.5 | 25 |
| 26 | 45 | 0 °C | 1 | 25 |
| 27 | 45 | 0 °C | 3 | 25 |
| 28 | 45 | 0 °C | 5 | 25 |
| 29 | 45 | 0 °C | 10 | 25 |
| 30 | 45 | 0 °C | 30 | 25 |
| 31 | 45 | 0 °C | 60 | 25 |
| 32 | 45 | 0 °C | 120 | 25 |

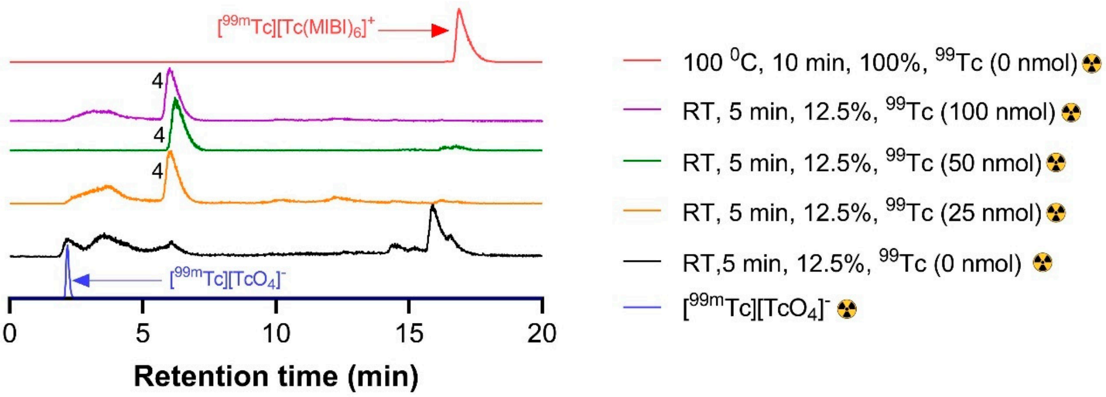


***Figure S 8*** *HPLC radiochromatograms (method 1, Table S2) of the reaction of [$^{99m}$Tc][$TcO_4$]$^-$ and several quantities of added carrier [$^{99}$Tc]pertechnetate (0 nmol blue, 25 nmol orange, 50 nmol green, 100 nmol purple) with a Technescan MIBI kit (12.5%) incubated at RT for 5 min. The control solution of [$^{99m}$Tc][$TcO_4$]$^-$ (blue) and the reaction performed at 100 °C (red) are also represented. The RCY of [$^{99/99m}$Tc]Tc- intermediate-4 increased with the concentration of $^{99}$Tc.*

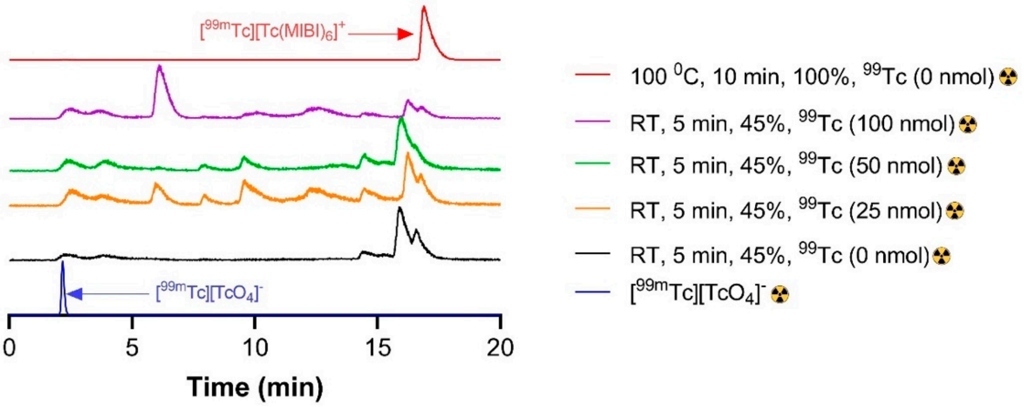


***Figure S 9*** *HPLC radiochromatograms (method 1, Table S2) of the reaction of fractionated kit (45%) with several quantities of added carrier $^{99}$Tc (0-20 µL, 0-100 nmol) and [$^{99m}$Tc][$TcO_4$]$^-$, incubated at RT for 5 min. The control solution of [$^{99m}$Tc][$TcO_4$]$^-$ (blue) and the reaction performed at 100 °C (red) are also represented. Increasing the concentration of carrier $^{99}$Tc resulted in a delay of the formation of the more lipophilic compounds.*

***Table S 7*** *Composition of bespoke MIBI kits containing variable amounts of [Cu(MIBI)4][BF4] and the standard amount of sodium citrate dihydrate, L-cysteine hydrochloride monohydrate, mannitol and SnCl2•2H2O.*

| Compound mass (mg/ µmol) | | | | | |
|---|---|---|---|---|---|
| **Kit** | **[Cu(MIBI)$_4$][BF$_4$]** | **Sodium Citrate Dihydrate** | **L-Cysteine Hydrochloride Monohydrate** | **Mannitol** | **$SnCl_2$•$2H_2O$** |
| **1** | **0/0** | 2.6/8.8 | 1.0/5.7 | 20.0/ 110.0 | 0.075/0.33 |
| **2** | **0.01/0.0166** | | | | |
| **3** | **0.1/0.166** | | | | |
| **4** | **1.0/1.66** | | | | |

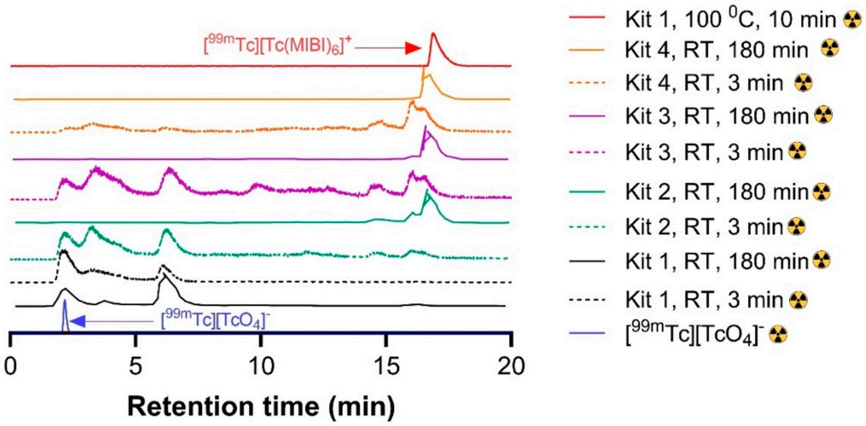


***Figure S 10*** *HPLC radiochromatograms (method 1, Table S2) reactions of bespoke kits containing varying amounts of [Cu(MIBI)$_4$][BF$_4$] with [$^{99m}$Tc][TcO$_4$]$^-$ prepared at RT for 3 min and 180 min. The control solution of [$^{99m}$Tc][TcO$_4$]$^-$ (blue) and the reaction performed at 100 °C (red) are also shown. Radiolabelled kits 1 (black), 2 (green), 3 (purple) and 4 (orange) contained 0 mg, 0.01 mg, 0.1 mg and 1 mg of [Cu(MIBI)$_4$][BF$_4$], respectively. The kit prepared without [Cu(MIBI)$_4$][BF$_4$] (kit 1) confirmed that [$^{99m}$Tc]Tc-intermediates eluted between 2.1 min and 6.3 min do not contain MIBI ligands. Increasing the amount of [Cu(MIBI)$_4$][BF$_4$] led to an increase in the formation of [$^{99m}$Tc][Tc(MIBI)$_6$]$^+$.*

***Table S 8*** *Bespoke kits prepared in-house with combinations of sodium citrate dihydrate, L-cysteine hydrochloride monohydrate, mannitol and SnCl$_2$•2H$_2$O.*

| Compound mass (mg/µmol) | | | | | |
|---|---|---|---|---|---|
| **Kit** | **[Cu(MIBI)$_4$][BF$_4$]** | **Sodium Citrate Dihydrate** | **L-Cysteine Hydrochloride Monohydrate** | **Mannitol** | **SnCl$_2$•2H$_2$O** |
| 5 | 0/0 | 2.6/8.8 | 0/0 | 0/0 | 0.075/0.33 |
| 6 | 0/0 | 0/0 | 1.0/5.7 | 20.0/110.0 | 0.075/0.33 |
| 7 | 0/0 | 0/0 | 1.0/5.7 | 0/0 | 0.075/0.33 |
| 8 | 0/0 | 0/0 | 0/0 | 20/110.0 | 0.075/0.33 |

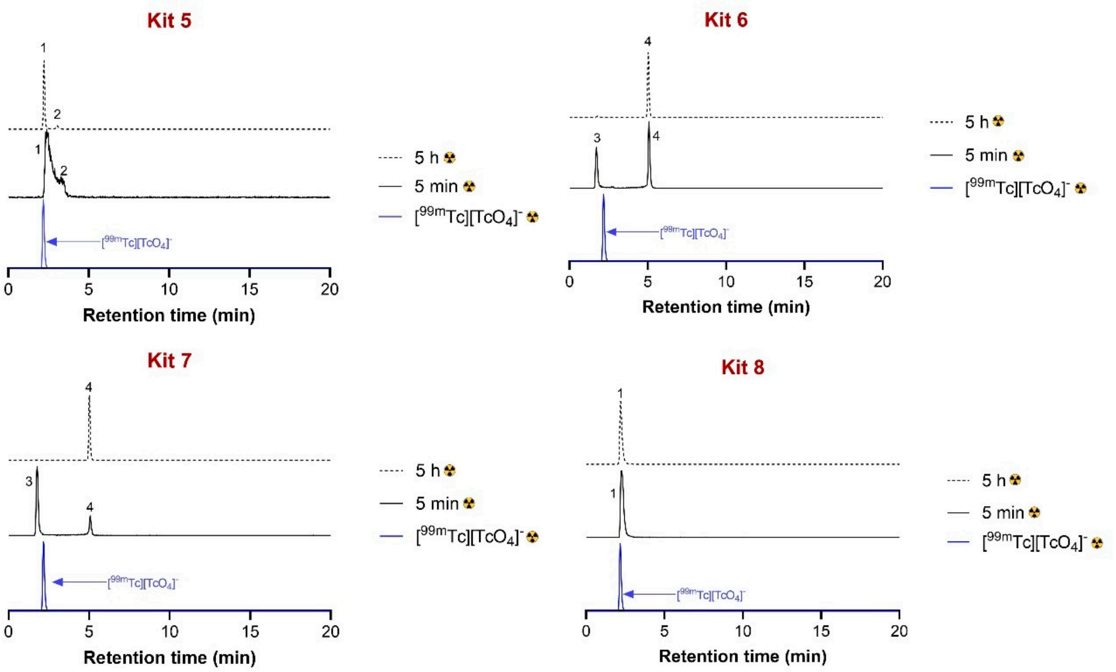


***Figure S 11*** *HPLC radiochromatograms (method 1, Table S2) of bespoke kits 5 (upper left), 6 (upper right), 7 (lower left) and 8 (lower right) radiolabelled with $[^{99m}Tc][TcO_4]^-$. A total of four $[^{99m}Tc]Tc$-intermediates eluting between 1.72 min and 5.02 min were detected. By omitting sodium citrate dihydrate and mannitol, a cysteine complex (kit 6-7, eluting at 5.02 min, peak 4) was potentially identified. By omitting L-cysteine and mannitol, a citrate complex (kit 5,eluting at 3.27 min, peak 2) was potentially identified.*

# Mannitol

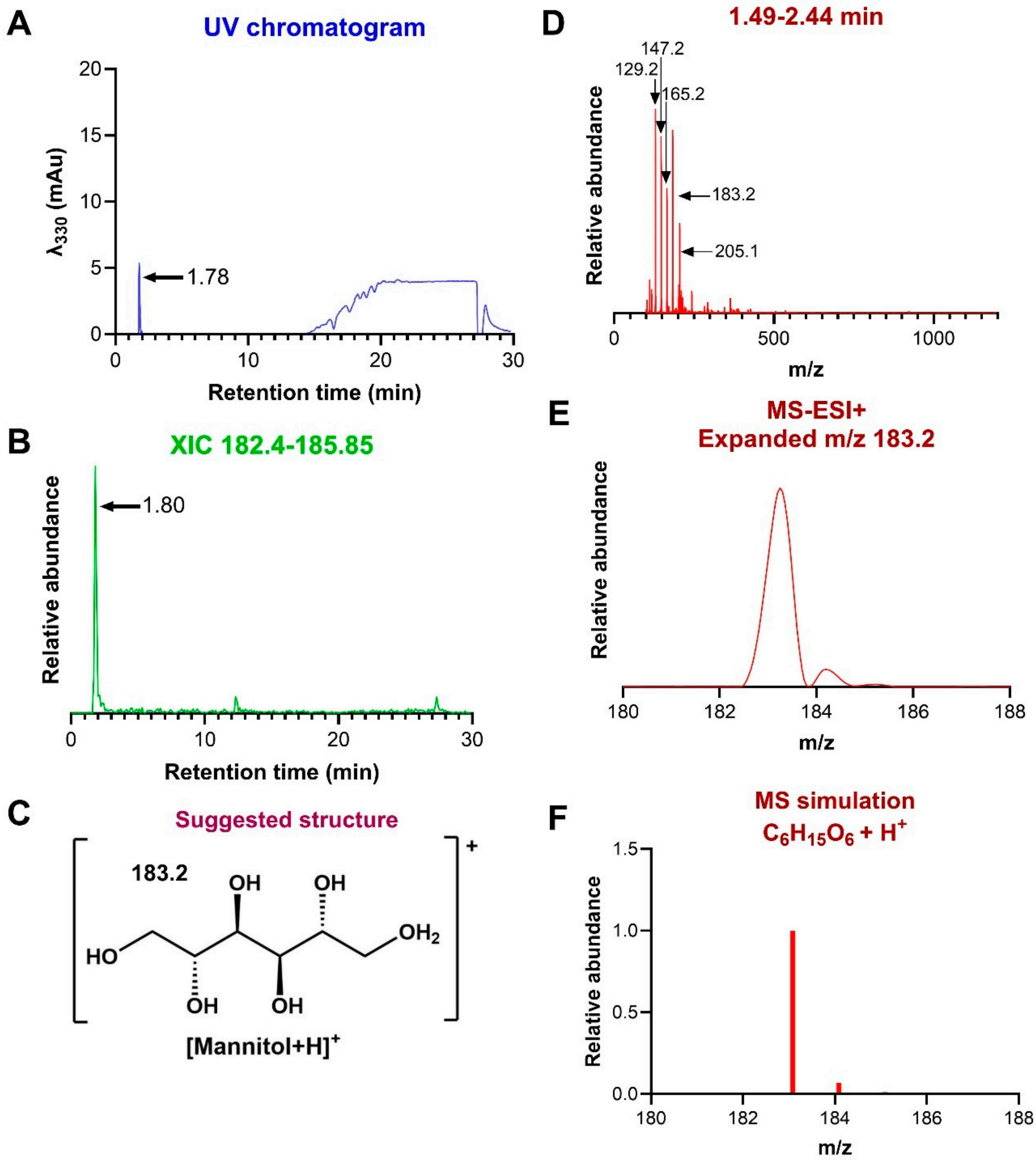


***Figure S 12*** *LCMS of mannitol.* ***A****: HPLC (method 1, Table S2), UV detection, 330 nm, showing elution at 1.78 min;* ***B****: Extracted ion chromatogram of m/z range 182.4-185.85, identifying molecular ion (mannitol+H)⁺ as peak eluting at 1.8 min;* ***C****: structure of (mannitol+H)⁺ molecular ion;* ***D****: mass spectrum of fraction eluting between 1.49 and 2.44 min, showing ions assignable to (mannitol+H)⁺ (183.2), (mannitol+Na)⁺ (205.1) and dehydrated mannitol derivatives ((mannitol-$H_2O$+H)⁺) (165.2), (mannitol-2$H_2O$+H)⁺ (147.2), (mannitol-3$H_2O$+H)⁺ (129.2));* ***E****: expanded view of m/z peak 183.2, corresponding to (mannitol+H)⁺;* ***F****: simulation of mass spectrum of (mannitol+H)⁺.*

# Citric acid

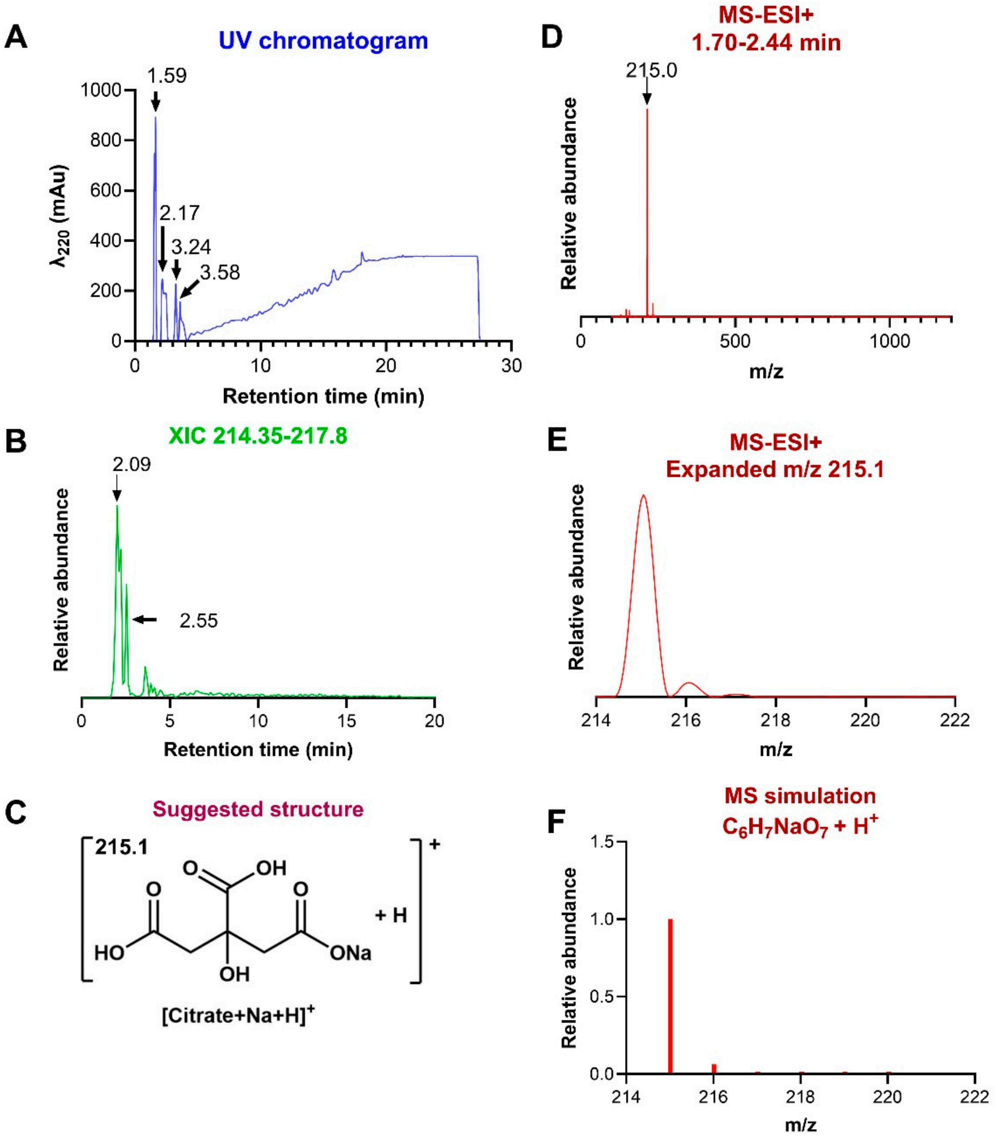


***Figure S 13*** *LCMS of citric acid.* ***A****: HPLC (method 1, Table S2) with UV detection 220 nm, showing complex elution profile 1.6-3.6 min;* ***B****: Extracted ion chromatogram of m/z 214.35-217.80, showing complex elution profile 2.1-2.6 min;* ***C****: structure of (sodium citrate+H)$^+$;* ***D****: mass spectrum of fraction eluting between 1.70 and 2.44 min, showing principal ion at m/z 215.0 assignable to (sodium citrate+H)$^+$;* ***E****: expanded view of m/z 215.0 peak;* ***F****: simulated mass spectrum of (sodium citrate+H)$^+$.*

# Cysteine

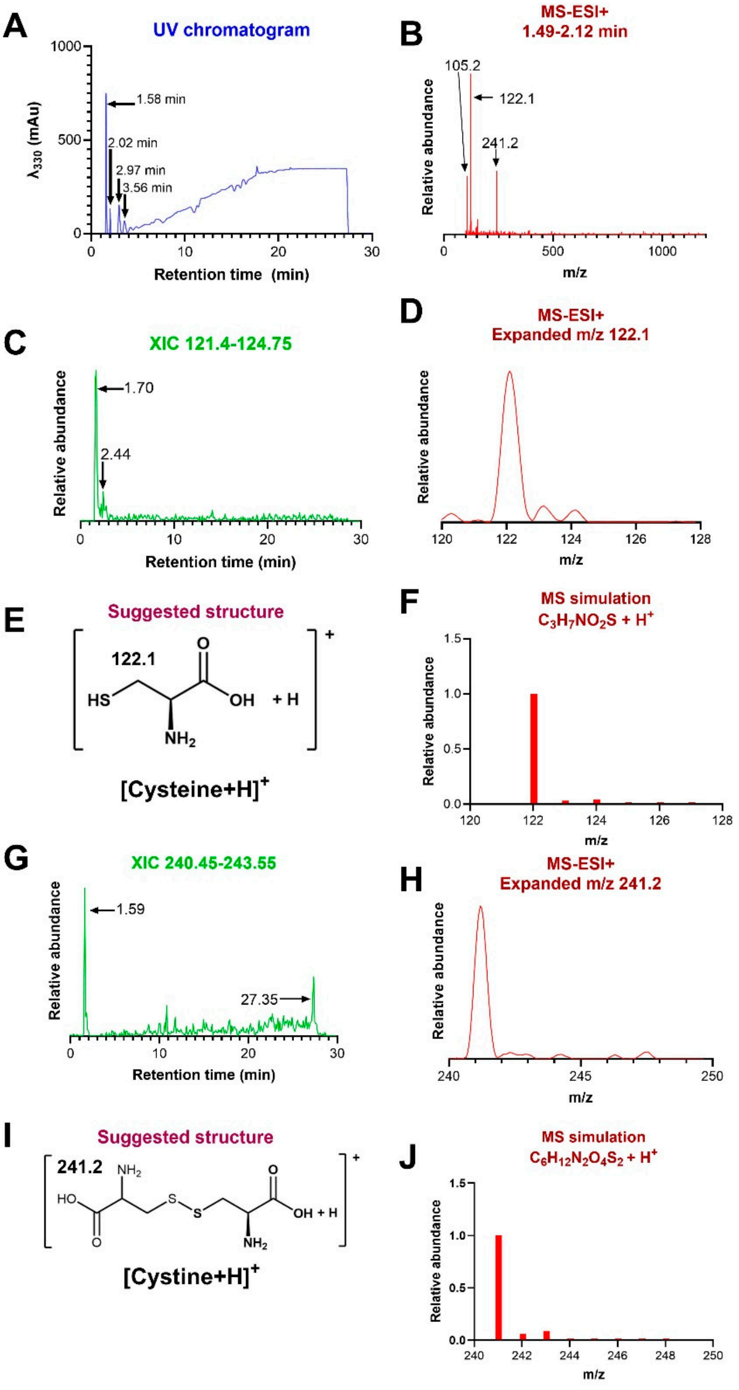


***Figure S 14*** *LCMS of cysteine.* ***A****: HPLC (method 1, UV detection 330 nm) showing complex elution profile 1.5-3.6 min;* ***B****: mass spectrum of fraction eluting 1.49-2.12 min, showing primary peak at m/z = 122.1 assignable to (cysteine+H)$^+$, and additional peaks at m/z = 105.2 assignable to (cysteine-$NH_3$+H)$^+$ and 241.2 assignable to (cystine+H)$^+$;* ***C****: extracted ion chromatogram of m/z range 121.40-124.75;* ***D****: expanded view of m/z = 122.1 ((cysteine+H)$^+$;* ***E****: structure of (cysteine+H)$^+$;* ***F****: simulated mass spectrum of (cysteine+H)$^+$:* ***G****: extracted ion chromatogram of m/z range 240.45-243.55 (assigned to (cystine+H)$^+$) identifying it as eluting at 1.58 min;* ***H****: expanded view of m/z = 241.2 (assigned to (cystine+H)$^+$;* ***I****: structure of (cystine+H)$^+$;* ***J****: simulated mass spectrum of (cystine+H)$^+$.*

## $[Cu(MIBI)_4][BF_4]$ (methanol-based mobile phase)

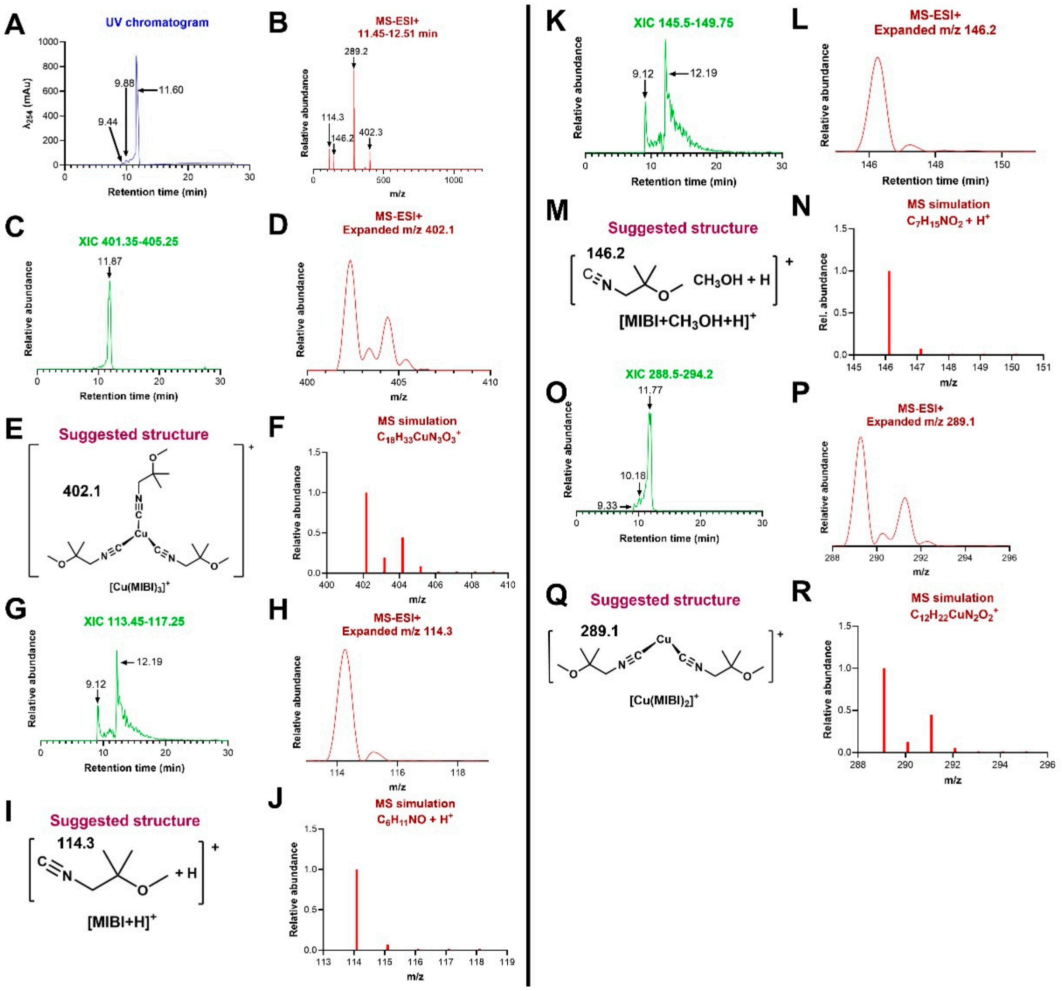

***Figure S 15*** *LCMS of $[Cu(MIBI)_4][BF_4]$ in methanol-based mobile phase.* ***A****: HPLC (method 1, Table S2) with UV detection 254 nm;* ***B****: mass spectrum of fraction eluting between 11.45 and 12.51 min ;* ***C****: extracted ion chromatogram of ions with m/z range 401.35-405.25;* ***D****: expanded view of peak with m/z = 402.1;* ***E****: Proposed structure of the ion giving rise to m/z = 402.18, $[Cu(MIBI)_3]^+$;* ***F****: simulated mass spectrum of $[Cu(MIBI)_3]^+$;* ***G****: extracted ion chromatogram of m/z 113.45-117.25;* ***H****: expanded view of m/z = 114.3;* ***I****: proposed structure of ion with m/z = 114.3, $(MIBI+H)^+$;* ***J****: simulated mass spectrum of $(MIBI+H)^+$;* ***K****: extracted ion chromatogram of ion with m/z = 146.2;* ***L****: expanded view of peak with m/z = 146.2;* ***M****: proposed structure of ion with m/z = 146.2: $(MIBI+MeOH+H)^+$;* ***N****: simulated mass spectrum of $(MIBI+MeOH+H)^+$;* ***O****: extracted ion chromatogram of ion with m/z = 289.1;* ***P****: expanded view of peak with m/z = 289.1;* ***Q****: proposed structure of ion with m/z = 289.1: $[Cu(MIBI)_2]^+$;* ***R****: simulated mass spectrum of $[Cu(MIBI)_2]^+$.*

## [Cu(MIBI)$_4$][BF$_4$] (acetonitrile-based mobile phase)

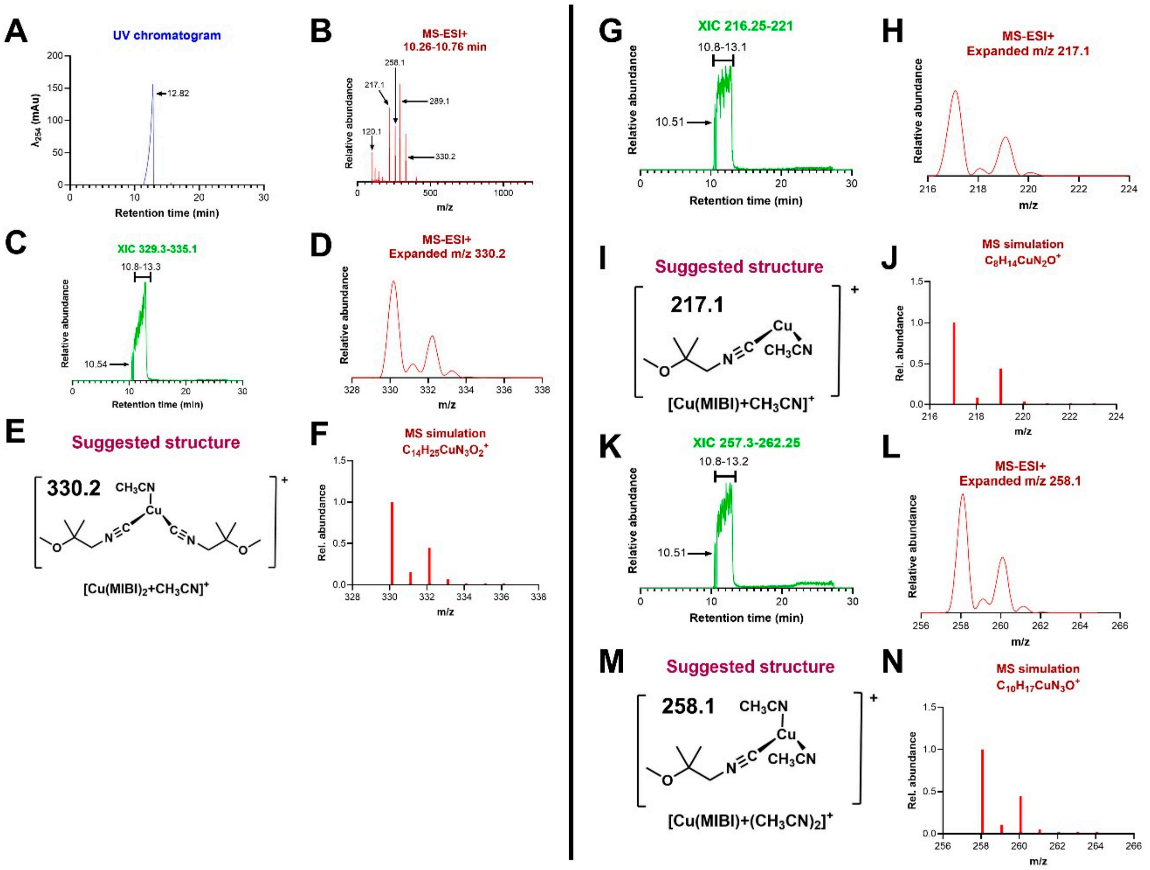


***Figure S 16*** *LCMS of [Cu(MIBI)$_4$][BF$_4$] in acetonitrile-based mobile phase.* ***A****: HPLC (method 3, Table S2) with UV detection, 254 nm;* ***B****: mass spectrum of fraction eluting between 10.26 and 10.76 min;* ***C****: extracted ion chromatogram of ions with m/z = 330.2;* ***D****: expanded view of peak with m/z = 330.2;* ***E****: proposed structure of ion with m/z = 330.2 ([Cu(MIBI)$_2$(MeCN)]$^+$);* ***F****: simulated mass spectrum of [Cu(MIBI)$_2$(MeCN)]$^+$;* ***G****: extracted ion chromatogram of ions with m/z = 217.1;* ***H****: expanded view of peak with m/z = 217.2;* ***I****; proposed structure of ion with m/z = 217.1: [Cu(MIBI)(MeCN)]$^+$;* ***J****: simulated mass spectrum of [Cu(MIBI)(MeCN)]$^+$;* ***K****: extracted ion chromatogram of ions with m/z = 258.1;* ***L****: expanded view of peak with m/z = 258.1;* ***M****: proposed structure of ion with m/z = 258.1: [Cu(MIBI)(MeCN)$_2$]$^+$;* ***N****: simulated mass spectrum of [Cu(MIBI)(MeCN)$_2$]$^+$.*

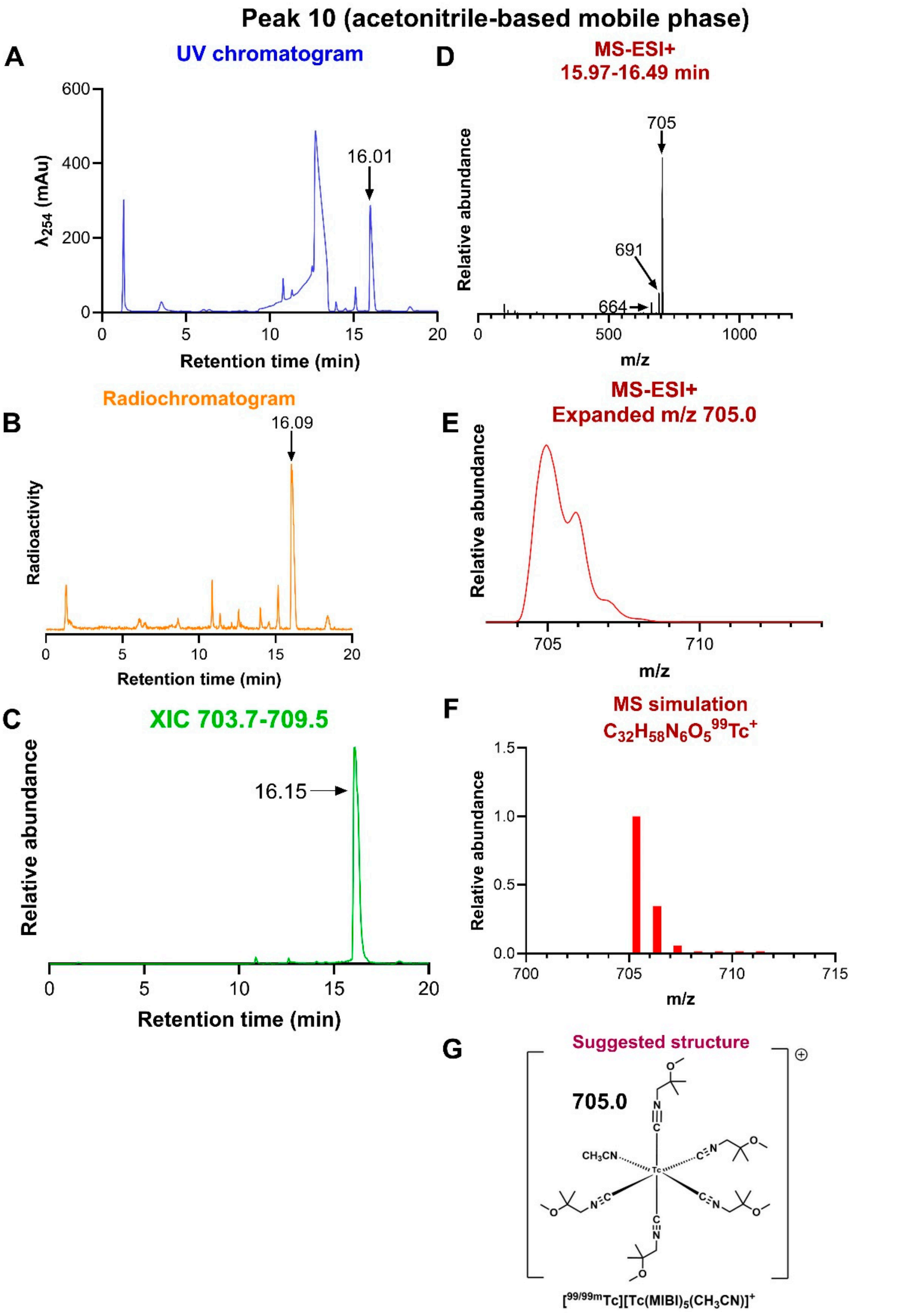


***Figure S 17*** *LCMS of peak 10 using acetonitrile-based mobile phase (method 3, Table S2).* ***A****: HPLC, UV detection 254 nm;* ***B****: radio-HPLC chromatogram, showing major peak eluting at 16.01 min;* ***C****: extracted ion chromatogram of ions with m/z = 705.0;* ***D****: mass spectrum of fraction eluting between 15.97 and 16.49 min;* ***E****: expanded view of peak with m/z = 705.0;* ***F****: simulated mass spectrum of [Tc(MIBI)$_5$(MeCN)]$^+$;* ***G****: proposed structure of ion with m/z = 705.0.*

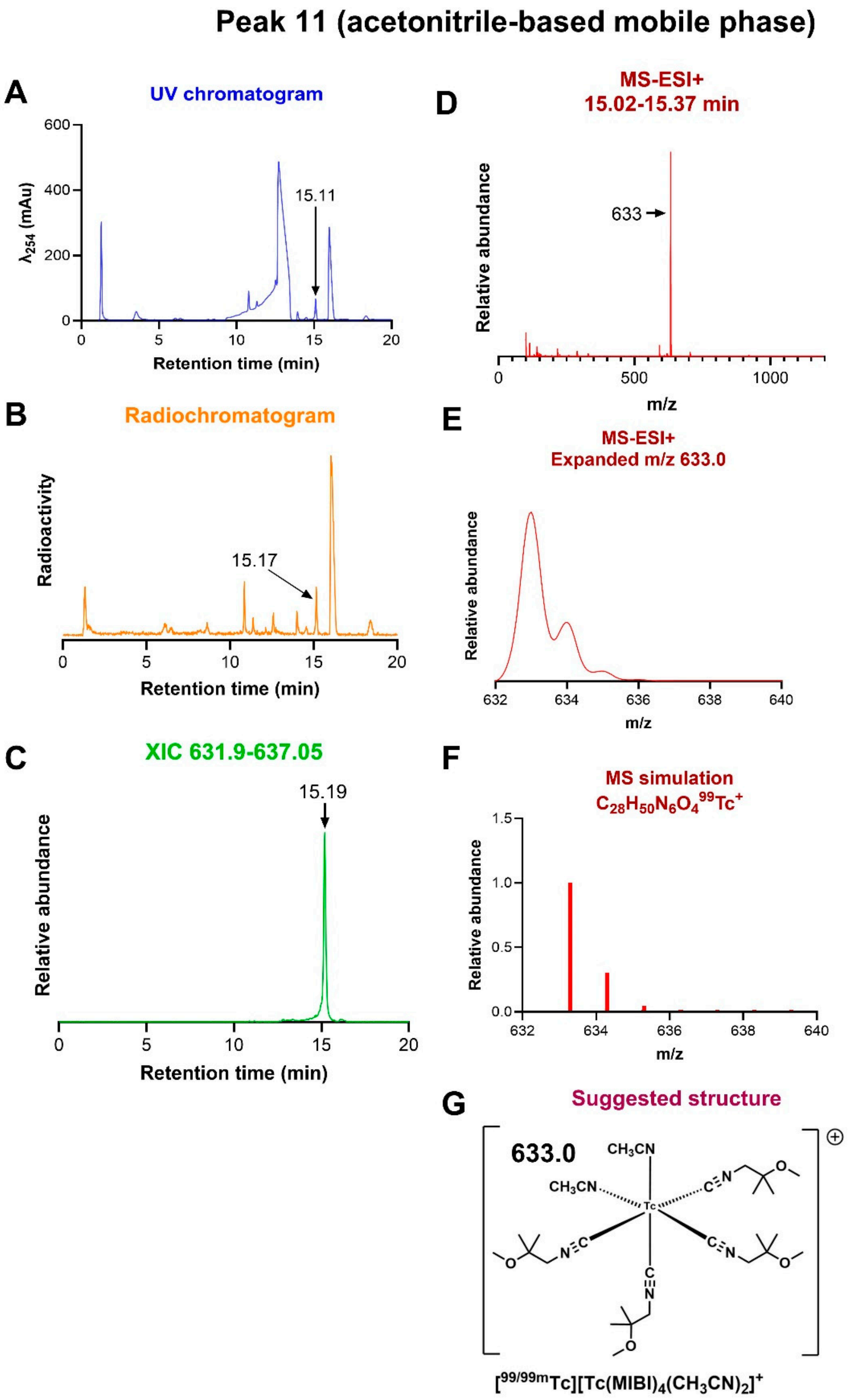


***Figure S 18*** *LCMS of peak 11 using acetonitrile-based mobile phase.* ***A****: HPLC (method 3, Table S2) with UV detection 254 nm;* ***B****: radiochromatogram showing peak eluting at 15.2 min (analogous to peak 11);* ***C****: extracted ion chromatogram of ions with m/z = 633.0 showing exclusive elution at 15.2 min;* ***D****: mass spectrum of fraction eluting between 15.02 and 15.37 min;* ***E****: expanded view of peak with m/z = 633.0;* ***F****: simulated mass spectrum of $[Tc(MIBI)_4(MeCN)_2]^+$;* ***G****: proposed structure of ion with observed m/z = 633.0.*

**Materials and Methods**

All reagents and consumables were purchased from Sigma Aldrich and Thermo Fisher Scientific unless specified otherwise. Saline was purchased from Severn Biotech (Worcestershire, UK). Radioactivity measurements were carried out using a dose calibrator Capintec CRC®-25R (Capintec Inc, USA). Size exclusion HPLC: Phenomenex size exclusion column (SEC)-s2000 column (5 µm, 145 Å, 300 x 7.8 mm, p.n. OOH-2145-K0), with a Phenomenex Securityguard GFC2000 4 x 3 mm guard cartridge (KJ0-4282, AJ0-4487).

*Effects of temperature and incubation time* were determined by reconstituting Technescan MIBI kits with generator eluate (typically 200 MBq), adjusted to 1.1 mL with saline, and incubating at RT, on ice (0 °C) or at 100 °C in a dry heater (in a dry heater (Mini Dry Bath, StarLab International GmbH Hamburg, Germany), sampling for TLC (methods 1 and 2, Table S1) and C18-HPLC (method 1, Table S2) analysis at intervals. Conditions are summarised in Table S3.

*Effects of fractionating* Technescan MIBI kits were determined by reconstituting with saline (1 mL) and increasing volumes of the resulting solution (62.5 µL, 125 µL, 450 and 1000 µL, corresponding to 6.25%, 12.5%, 45%, and 100% of the total kit (Table S4), were combined with generator eluate (4.23-5.56 MBq). After incubation at RT for 5 min the solutions were analysed by C18 HPLC (method 1, Table S2). The reaction of the unfractionated kit incubated at 100 °C for 10 min was used to form $[^{99m}Tc][Tc(MIBI)_6]^+$ as the reference compound.

*Effects of reducing the quantity of individual kit components*, relative to other constituents, were determined with bespoke kits prepared in-house, with varying masses of $[Cu(MIBI)_4][BF_4]$ (Kits 1-4, Table S7), sodium citrate dihydrate, L-cysteine hydrochloride monohydrate and mannitol (kits 5-8, Table S8) each with the standard quantities of the other constituents. Ingredients were dissolved in nitrogen-purged water, the solutions  adjusted to pH 5.4-5.6 with 1M NaOH and 1M HCl and dispensed in 1 mL fractions into nitrogen-filled vials, frozen in liquid nitrogen and freeze-dried with a freeze-drier (Edwards Modulyo) for 24 h. The lyophilised kits were purged with argon gas for storage. These kits, designated 1-8, were reconstituted with generator eluate (155.0 MBq-185.0 MBq, 296-400 µL, adjusted to 1.1 mL with saline) and incubated at RT for up to 5 h, analysing at intervals by TLC (methods 1 and 2, Table S1), and C18-HPLC (method 1, Table S2).

*Effect of carrier $^{99}$Tc concentration* on the profile of $[^{99m}Tc]$Tc-intermediates, to aid later identification by LCMS,  was determined by adding $^{99}$Tc in the form of ammonium pertechnetate (purchased from Amersham International, UK in 1988, dissolved in water 1 mM and filtered). A Technescan MIBI kit was reconstituted with saline (1 mL). Solutions of generator eluate (ca. 5 MBq) and $^{99}$Tc (5 µL - 20 µL; 25-100 nmol) were combined and the solution added to increasing volumes of the pre-diluted kit solutions (125 µL, 450 µL and 1000 µL, corresponded to 12.5%, 45% and 100% of kit components, respectively). The mixtures were vortexed and incubated at RT for up to 24 h, sampling for C18-HPLC (method 1, Table 2) at intervals. A total of 32 reactions were performed (Table S6).

*Isolation of intermediates.* Suspecting that some of the more persistent intermediates in the HPLC radiochromatogram might have sufficient kinetic stability to be isolated for further analysis, attempts were made to separate them by HPLC (methods 1 and the extended gradient of method 2, an extended water/methanol/formic acid gradient to enhance resolution of the more lipophilic species) and assess their stability by re-analysing the purified fractions using the same HPLC gradient. Intermediates 4 (Fig. S19), 10 (Fig. S20), 11 (Fig. S21) and 12 (Fig. S22) were analysed in this way. Of these, only intermediates 4 (elution time ca. 6 min, method 1) and 12 (elution time ca. 32 min, method 2) showed the same elution time on re-analysis and were thus deemed to be sufficiently stable to be isolated. Intermediate 10 (elution time ca. 26 min, method 2) was completely degraded to a more hydrophilic species (elution time ca. 14 min) and intermediate 11 (elution time ca. 31 min, method 2) was partially (ca. 40%) degraded to another more hydrophilic species (elution time 18 min).

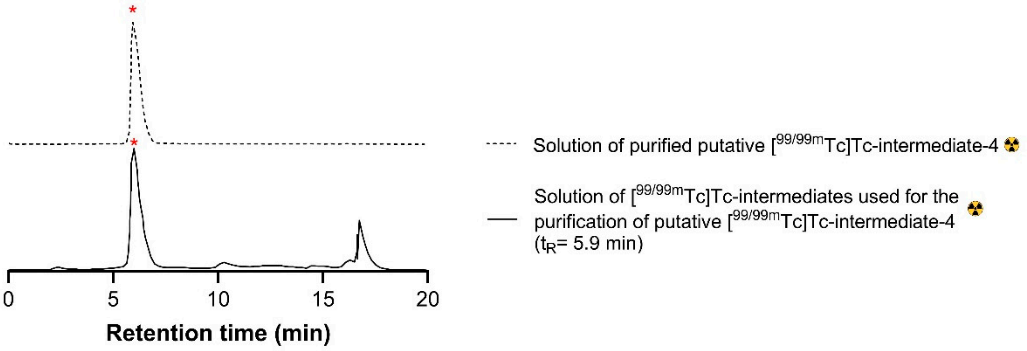


***Figure S 19*** *HPLC radiochromatograms of the crude reaction of kit (12.5%) with [$^{99m}$Tc][$TcO_4$]$^-$ and carrier $^{99}$Tc (50 nmol) to produce putative [$^{99/99m}$Tc]Tc-intermediate-4 (asterisk, bottom). The HPLC-purified putative [$^{99/99m}$Tc]Tc-intermediate-4 (asterisk) was represented at the top. [$^{99/99m}$Tc]Tc-intermediate-4 remained intact after purification.*

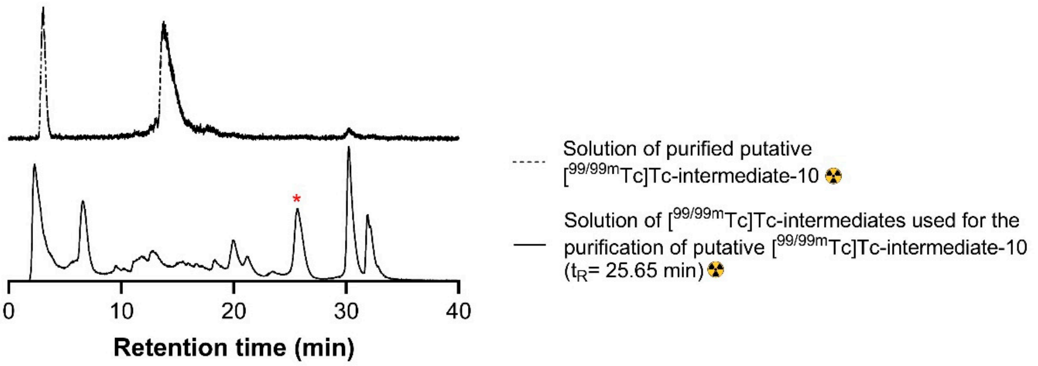


**Figure S 20** *HPLC radiochromatograms (method 2, Table S2) of the crude reaction of kit (45%) with [$^{99m}$Tc][$TcO_4$]$^-$ and carrier $^{99}$Tc (50 nmol)) used to purify putative [$^{99/99m}$Tc]Tc-intermediate-10 (bottom, asterisk). The putative [$^{99/99m}$Tc]Tc-intermediate-10 eluted originally at 25.7 min (bottom, red asterisk). However, after purification, the purified [$^{99m}$Tc]Tc-intermediate-10 showed a split into two main peaks, which eluted at 3.1 min and 13.8 min (top).*

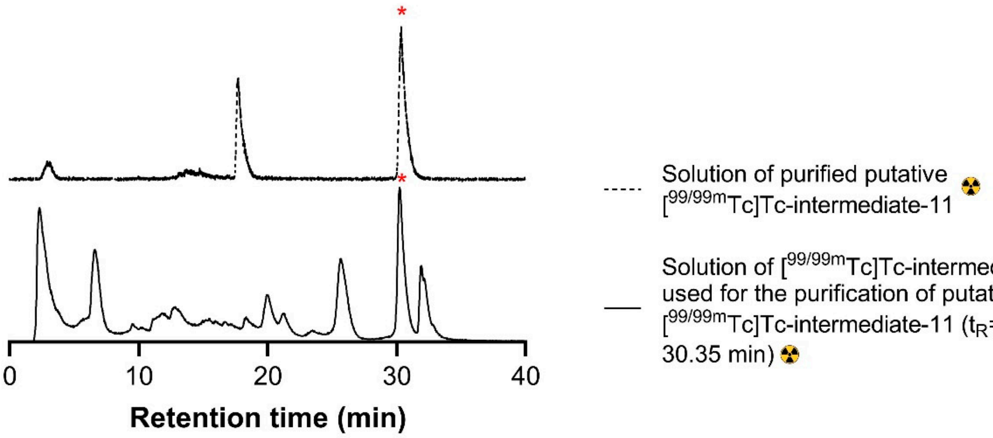


**Figure S 21** *HPLC radiochromatograms (method 2, table S2) of the crude reaction of kit (45%) (bottom) with [$^{99m}$Tc][$TcO_4$]$^-$ and carrier $^{99}$Tc (50 nmol) used to purify the putative [$^{99/99m}$Tc]Tc-intermediate-11 (bottom, asterisk), and solution of the purified putative [$^{99/99m}$Tc]Tc-intermediate-11 (top). The purified putative [$^{99/99m}$Tc]Tc-intermediate-11 (top, asterisk) eluted at 30.35 min (53.4%), accompanied by additional earlier-eluting peaks.*

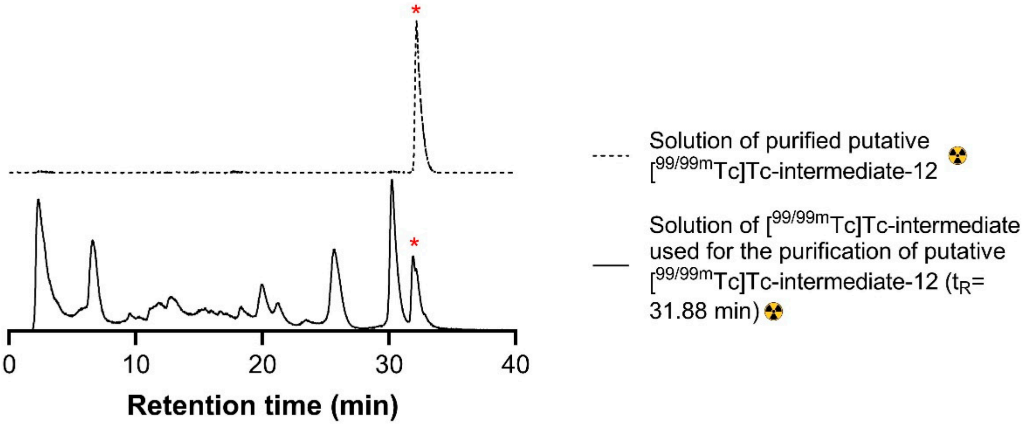


***Figure S 22*** *HPLC radiochromatograms (method 2, Table S2) of the crude reaction of kit (45%) with [$^{99m}$Tc][$TcO_4$]$^-$ and carrier $^{99}$Tc (50 nmol) used to purify putative [$^{99/99m}$Tc]Tc-intermediate-12* **(***bottom, asterisk), and solution of the purified putative [$^{99/99m}$Tc]Tc-intermediate-12 (top). This compound (top, asterisk) was stable after purification.*

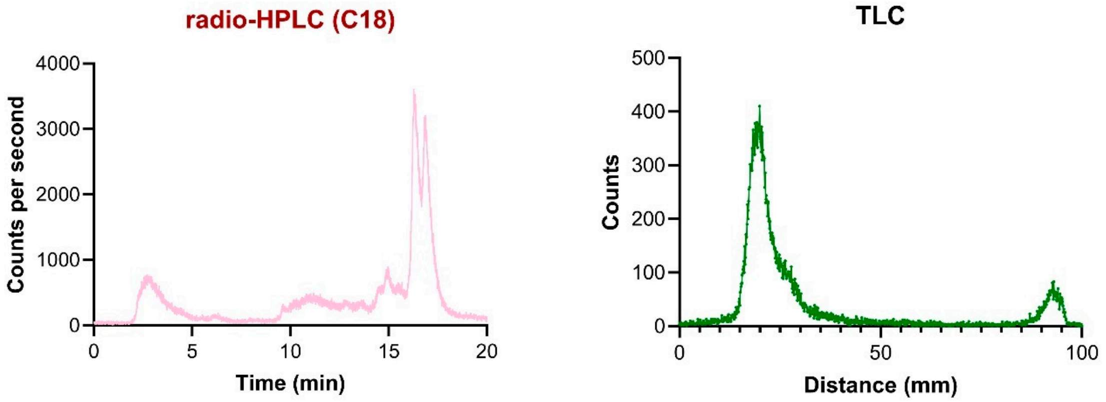


***Figure S 23*** *Exemplar TLC (method 2A, left) and HPLC (method 1, right) of Technescan MIBI kit incubated on ice for 60 min. The apparent radiochemical purity of [$^{99m}$]Tc-Sestamibi quantified by TLC (Rf = 0, ITLC-SG/saline) (right)) was >90%, yet HPLC showed the % of [$^{99m}$]Tc-Sestamibi to be < 32%.*

## LCMS of [$^{99m}$Tc]Tc-intermediates

The results of the foregoing experiments were used to identify sets of conditions to optimise formation of selected radioactive peaks in the radiochromatograms. A summary of the chosen reaction conditions and their "target" peaks is provided in Table S4.
